# Mortise-tenon–shaped memristors for scientific computing

**DOI:** 10.1126/sciadv.adu3309

**Published:** 2025-04-30

**Authors:** Weiqi Dang, Yu Shen, Wei Wei, Chen Pan, Fanqiang Chen, Gong-Jie Ruan, Yan Luo, Ying Guo, Qiuyang Tan, Jingwen Shi, Xing-Jian Yangdong, Sicheng Chen, Cong Wang, Yongqin Xie, Zai-Zheng Yang, Pengfei Wang, Shuang Wang, Li Zhong, Shaobo Cheng, Chao Zhu, Bin Cheng, Shi-Jun Liang, Feng Miao

**Affiliations:** ^1^ Institute of Brain-Inspired Intelligence, National Laboratory of Solid State Microstructures, School of Physics, Collaborative Innovation Center of Advanced Microstructures, Nanjing University, Jiangsu Physical Science Research Center, Nanjing 210093, China.; ^2^ Institute of Interdisciplinary Physical Sciences, School of Physics, Nanjing University of Science and Technology, Nanjing 210094, China.; ^3^ SEU-FEI Nano-Pico Center, Key Laboratory of MEMS of Ministry of Education, School of Integrated Circuits, Southeast University, Nanjing 210096, China.; ^4^ Henan Key Laboratory of Diamond Optoelectronic Materials and Devices, Key Laboratory of Material Physics, Ministry of Education, School of Physics and Microelectronics, Zhengzhou University, Zhengzhou 450052, China.

## Abstract

In-memory computing hardware based on memristors has emerged as a promising option for scientific computing due to its large-scale parallel data processing capability. However, the nonuniformity issue of the memristors renders the practical deployment of in-memory computing hardware complex, requiring peripheral circuits to ensure the accuracy of scientific computing, thereby resulting in increased power consumption. Here, we present a mortise-tenon–shaped (MTS) memristor with ultrahigh uniformity by introducing a mortise-shaped h-BN flake on the HfO_2_ switching layer. The MTS memristor exhibits ultrasmall cycle-to-cycle (~2.5%) and device-to-device (~6.9%) variations compared to the HfO_2_ memristor without the MTS structure. Furthermore, we use the MTS memristors to build a partial differential equation solver and demonstrate a convergence speed of solving the Poisson equation five times faster than the solver based on the traditional HfO_2_ memristors. This work provides a promising approach for notably reducing the hardware resources required for fast and high-accuracy scientific computing.

## Teaser

Traditional Chinese architecture inspires the design of mortise-and-tenon high-uniformity memristors for scientific computing.

## INTRODUCTION

Scientific computing is essential for solving complex physical problems in various fields, including weather forecasting, fluid dynamics simulations, and astrophysical modeling (*1*–*3*). In scientific computing, solving partial differential equations (PDEs) is essential for addressing most problems. A critical factor in efficiently implementing this process is the ability to perform large-scale matrix-vector multiplications (MVMs). This requires hardware with highly parallel information processing capabilities to handle a large number of parameters (*4*–*12*). The emerging in-memory computing technology enables highly parallel information processing directly within the memory, making it an ideal hardware platform for implementing MVM (*13*–*21*). Memristor, as one of the emerging nonvolatile devices, provides a promising hardware solution to the in-memory computing. Memristors offer higher integration density and lower power consumption than other memory technologies (*22*–*32*). The PDE solvers based on the memristors can provide faster and more accurate solutions compared to conventional silicon-based device technologies (*33*–*39*). However, programming the memristors suffers from nonuniformity issues. Existing technologies necessitate the use of peripheral circuits to improve the weight writing precision of memristors (*40*–*44*). This approach not only demands additional chip area but also results in considerable energy consumption. Alternatively, enhancing the programming uniformity of memristors at the device level presents a promising avenue for improving computation accuracy without an additional energy budget.

In this work, we present a high-uniformity memristor based on a mortise-tenon–shaped (MTS) architecture and demonstrate a low-power PDE solver using the proposed memristors. We fabricated the memristor based on a top electrode/h-BN/HfO_2_/bottom electrode heterostructure. In the h-BN/HfO_2_ heterostructure, we created a mortise-shaped structure by etching a hole within the h-BN layer. A top electrode with a tenon-shaped structure was then prepared on the h-BN, allowing it to make a direct contact with the HfO_2_ layer through the mortise-shaped structure. The proposed architecture ensures highly uniform switching behavior within the designated tenon-and-mortise region, endowing the MTS memristor with exceptional fundamental properties, including high endurance (>10^9^ cycles), stable retention (>10^4^ s), and fast switching speed (~4.2 ns). Furthermore, this MTS architecture enables memristors to exhibit notably improved programming uniformity. The cycle-to-cycle and device-to-device variation can be reduced down to 2.5 and 6.9%, respectively. Moreover, we demonstrate that the PDE based on the MTS memristors achieves a convergence speed of solving the Poisson equation more than five times faster than the solvers based on traditional HfO_2_ memristors. Our findings provide a promising strategy for developing high-uniformity memristors, paving the way for next-generation scientific computing platforms.

## RESULTS

### The device structure of the MTS memristor

**Fig. 1.**
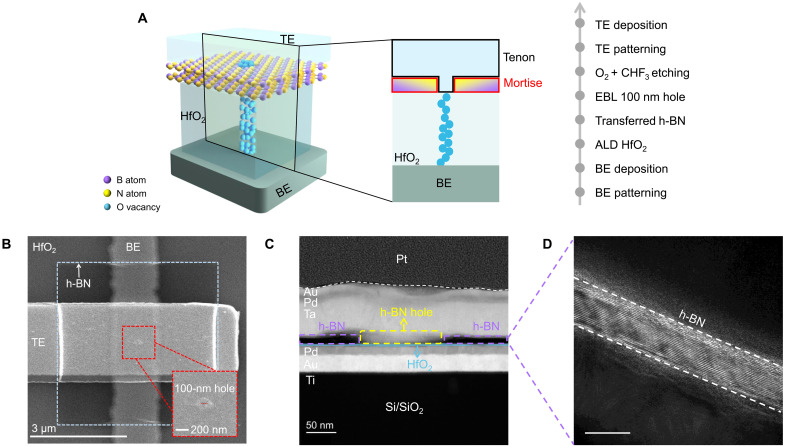
Device structure of the MTS memristor. (**A**) Device schematic and fabrication process flow of the MTS memristor. (**B**) SEM image of the MTS memristor. The blue dashed box represents h-BN. Scale bar, 3 μm. The inset shows the MTS region, with a diameter of ~100 nm. (**C**) HAADF-STEM characteristics of the MTS memristor. The yellow dashed box indicates the Ta metal electrode with a tenon-shaped structure making contact with the HfO_2_ switching layer through the mortise of h-BN, and the purple dashed box represents the unetched h-BN. Scale bar, 50 nm. (**D**) The unetched h-BN area in (C) is enlarged, showing that the thickness of the h-BN is ~8 nm. Scale bar, 10 nm.

Figure 1 illustrates the device structure of a typical proposed MTS memristor, which draws inspiration from the interlocking structures used in ancient Chinese architecture. Specifically, in the h-BN/HfO_2_ heterostructure, a mortise-shaped structure was created by etching the h-BN to form a hole structure. A top electrode with a tenon-shaped structure was then deposited on the top of the h-BN/HfO_2_ heterostructure (as shown in Fig. 1A). Compared to graphene (*45*, *46*), h-BN offers not only ion impermeability but also excellent insulating properties. These characteristics effectively shield the electric field outside the MTS region, confining ion migration within the switching layer to occur exclusively beneath the MTS region. Consequently, conductive filaments (CFs) are formed only within the switching layer under the MTS region, eliminating positional randomness and notably enhancing device uniformity. The main fabrication process flow of the MTS memristor is shown on the right side of Fig. 1A, and the detailed device fabrication process is provided in fig. S1. The dimension characteristics of a typical MTS memristor can be observed in Fig. 1B through a scanning electron microscope (SEM) image. The thickness of HfO_2_ is about 3 nm (see fig. S2). The h-BN flake is featured with a size of 5 μm by 5 μm (as shown in the blue dashed box in Fig. 1B) and an MTS region with a diameter of ~100 nm, as labeled by the red dashed box inset of Fig. 1B. We further characterized the MTS memristor by using the cross-sectional high-angle annular dark-field scanning transmission electron microscopy (HAADF-STEM), with the corresponding results shown in Fig. 1C. We observe a clear interruption spanning ~100 nm in the h-BN flake, where Ta metal with a tenon-shaped structure was deposited to make direct contact with the HfO_2_ switching layer at the MTS region. The multilayer h-BN is clearly visible with a thickness of ~8 nm, as evidenced by the magnified high-resolution transmission electron microscope images shown in Fig. 1D. The false-color STEM image of the MTS memristor (see fig. S3), with magnification and atomic images of h-BN at different interface positions, further proves that the fabricated memristor has a typical MTS structure shown in Fig. 1A.

### High-uniformity performance of the MTS memristors

**Fig. 2.**
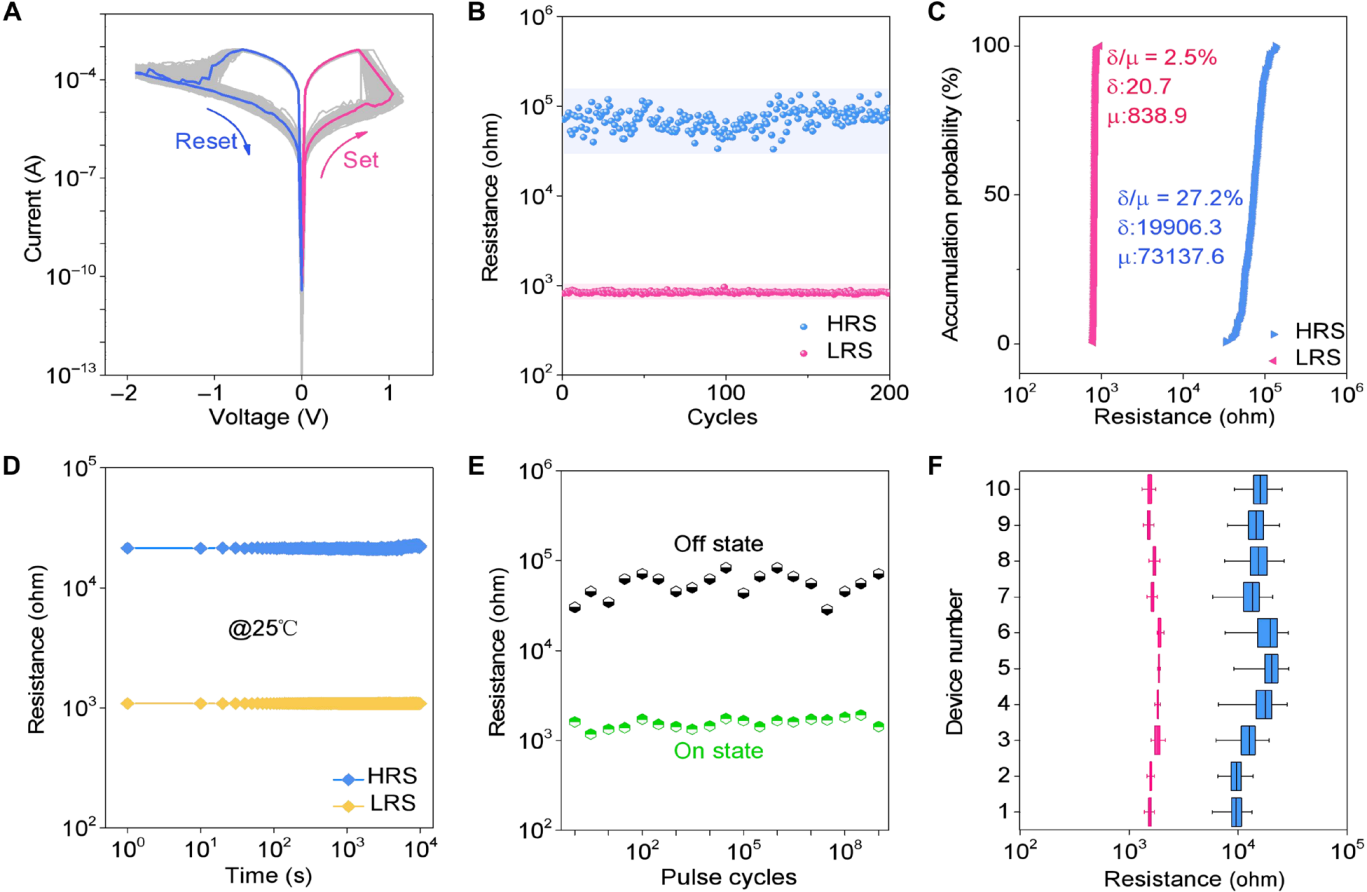
Switching performance of the MTS memristor. (**A**) A typical *I-V* measurement was conducted for 200 cycles on the MTS memristor. (**B**) The values of LRS and HRS changed continuously during 200 cycles of operation at *V*_read_ = 0.1 V. (**C**) The cumulative probability plot of LRS and HRS for the MTS memristor over 200 cycles demonstrates an ultralow variation of the LRS (~2.5%). (**D**) The retention of the MTS memristor at room temperature was measured at *V*_read_ = 0.1 V. (**E**) The pulse endurance characteristics of the MTS memristor (SET pulse, 1.5 V/2 μs; RESET pulse, −1.7 V/2 μs). (**F**) The box-and-whisker plot shows the distribution of LRS and HRS across 200 switching cycles for 10 MTS memristors. The red boxes and blue boxes represent LRS and HRS, respectively.

We characterized the switching performance of the MTS memristor through standard two-terminal electrical measurements. Figure 2A shows the switching curves of a typical MTS for 200 cycles. The MTS memristor exhibits stable and forming-free bipolar switching characteristics. The initial set and reset operations (red curve and blue curve in Fig. 2A) are consistent with subsequent switching curves, indicating that no drastic electroforming process has occurred. This forming-free behavior mainly arises from the use of a thinner HfO_2_ switching layer in the memristors, which eliminates the need to form CFs by using an overshoot voltage (*47*). Figure 2B shows the measured low resistance state (LRS) and high resistance state (HRS) of the MTS memristor at *V*_read_ = 0.1 V. Both LRS and HRS exhibit uniform distribution characteristics. We quantify the uniformity of the LRS and the HRS by using the coefficient of variation, *C*_v_ = σ/μ, where σ is the SD of resistance values over multiple cycles and μ is the average resistance value over multiple cycles (*48*). The *C*_v-L_ of the LRS is ultralow and reaches ~2.5%, which is crucial for updating weights accurately in scientific computing, while *C*_v-H_ of the HRS is about ~27.2%. Besides, we also quantified the uniformity of *V*_set_ and *V*_reset_, with the results shown in fig. S4, which shows a *C*_v_ of 11.7% for *V*_set_ and 2.9% for *V*_reset_. Note that a thinner h-BN flake (~4.6 nm) can also be used to fabricate the MTS memristors, and their performance remains consistent with that of the MTS device using 8-nm h-BN (fig. S5). In addition, the MTS memristor exhibits excellent characteristics in retention and pulse endurance. In Fig. 2D, both the LRS and the HRS states demonstrate favorable retention performance of >10^4^ s at room temperature without degradation, indicating stable nonvolatile memory. In terms of pulse endurance, the MTS memristor maintains a distinguishable LRS state and HRS state even after cycling through >109 pulse cycles, meeting the commercial require-ment of resistive random-access memory (i.e., pulse endurance >10^6^) (*49*). Besides, the MTS memristor also exhibits a turn-on characteristic of 4.2 ns (fig. S6). These figures of merit (retention, pulse endurance, and switch speed) make the MTS memristor suitable for implementing scientific computing applications.

We show that the performance of the MTS memristor is drastically improved over the traditional memristor without the MTS structure. To prove the advantage of the MTS memristor, we fabricated a HfO₂ memristor without the MTS structure and then carried out experimental measurements of cycle-to-cycle uniformity, pulse endurance, retention, switching speed, and on/off ratio, with the corresponding results shown in fig. S7. We compared the performance of the MTS memristor with the corresponding results presented in fig. S7 as the radar chart presented in fig. S8. The chart demonstrates that the introduction of the MTS structure into traditional memristors can substantially enhance the overall performance of the devices. The cycle-to-cycle variation of the LRS can be reduced from 30.3% for the traditional memristor to 2.5% for the MTS memristor. With the use of the MTS structure, the cycle-to-cycle variation of the HRS is reduced from 62.4 to 27.2%, and other figures of merit are also dramatically improved. We further made a comparison between the MTS memristor and those HfO₂-based memristors without the MTS structure (table S1), highlighting the smallest LRS variation of the MTS memristor proposed in this work.

The MTS memristors exhibit exceptional device-to-device uniformity and enable the reliable combination of multiple devices for precise weight representation, which is crucial for implementing scientific computing with memristors. To assess the device-to-device uniformity, we performed multi-cycle current-voltage relation sweep on 10 MTS memristors and then analyzed the resistance variations of these memristors (fig. S9). The resistance distribution of these 10 devices shows slight differences in resistance values, with an average variation of 6.9% for LRS and 26.0% for HRS. As a control experiment, we also evaluated the device-to-device uniformity of 10 HfO_2_ memristors without the MTS structure (fig. S10 and fig. S11) and compared these experimental results with the corresponding statistical results of the MTS memristors (tables S2 and S3). The comparison clearly demonstrates that the memristors based on HfO_2_ without the MTS structure show a larger variation of the LRS (on average 19.9%) and the HRS (on average 290.4%). These results further highlight the notable benefits of introducing the MTS structure into traditional memristors.

### The working mechanism of the MTS memristor

**Fig. 3.**
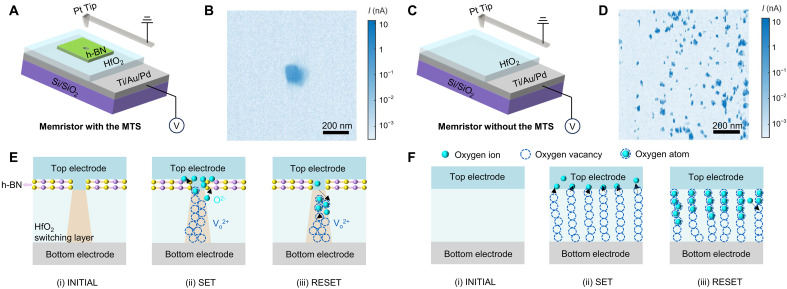
Distribution characteristics of CFs in the MTS memristor. (**A**) Schematic illustration of C-AFM measurement in the MTS memristor. (**B**) The current map measured by C-AFM of the MTS memristor demonstrates that CFs are likely to form only in the MTS region. Scale bar, 200 nm. (**C**) Schematic of C-AFM experiments in the control HfO_2_ (3-nm-thick) memristor. (**D**) The current map measured by C-AFM of the memristor without the MTS structure shows that CFs have the potential to form randomly throughout the entire switching layer. Scale bar, 200 nm. (**E**) Schematics of the MTS memristor in the INITIAL state, SET process, and RESET process, respectively. (**F**) Schematics of the control memristor without the MTS structure in the INITIAL state, SET process, and RESET process, respectively.

We investigate the operating mechanism of the MTS memristor by using the conductive atomic force microscopy (C-AFM) to characterize the microscale spatial distribution of the conductive region within the switching layer. Figure 3A shows a schematic diagram of the C-AFM setup used for characterizing the MTS memristor. Detailed processes of carrying out C-AFM measurements are outlined in Materials and Methods. Figure 3B shows the corresponding measured current map of the MTS memristor by using the C-AFM measurements. We observe a conductive region approximately 100 nm in width, confirming the formation of CFs only at the MTS region, which is consistent with the schematic depicted in Fig. 1A. Note that the measured current level is lower than that of the device shown in Fig. 2. This discrepancy is due to the use of a Pt tip as the top electrode in the C-AFM measurement, which has a higher contact resistance compared to the top electrode of the device shown in Fig. 1A. In sharp contrast, for the HfO_2_ memristor without the MTS structure (Fig. 3C), we observe the randomly distributed conductive regions throughout the entire current map (Fig. 3D). These findings indicate that the use of the MTS structure allows confining the switching behavior of the device within a fixed region during each cycle, thus achieving improvement of device uniformity.

On the basis of the comparative experimental results above, we propose the following mechanism for the MTS memristor, as schematically shown in Fig. 3E. For the MTS memristor, the INITIAL state (i) in Fig. 3E shows that the electrical contact between the tenon-shaped top electrode metal and the HfO_2_ switching layer is confined to beneath the MTS region, as indicated by the orange trapezoid in the HfO_2_ layer. Upon applying a positive voltage to the top electrode while grounding the bottom electrode, electrons are gained by the O atoms at the interface between HfO_2_ and the bottom electrode, causing their transformation into O^2−^. These O^2−^ ions migrate toward the top electrode, leading to the creation of oxygen vacancies within the switching layer. Once sufficient oxygen vacancies are generated to connect the top and bottom electrodes, CFs are fully formed within the switching layer and the device is turned on. This process is referred to as the SET process [(ii) in Fig. 3E] Conversely, the application of a negative voltage to the top electrode induces the migration of O^2−^ ions stored in the top electrode toward the bottom electrode. This migration results in the recombination of O^2−^ ions with oxygen vacancies present in the HfO_2_ layer, thereby reconstituting O atoms. Consequently, the CF formed by the original oxygen vacancies is disrupted, turning the device off, which is referred to as the RESET process [(iii) in Fig. 3E]. In general, the ion movement within the switching layer of the MTS memristor is effectively confined within the MTS region, while the undesired randomness of CFs is mitigated by the insulating properties of mortise-shaped h-BN. This confinement ensures reproducible switching behavior in terms of electrical performance and guarantees uniformity in cycle-to-cycle and device-to-device operations. Conversely, in HfO_2_ memristors without the MTS structure (depicted in Fig. 3F), CFs are randomly distributed across the entire crossbar region, resulting in inferior switching performance compared to the MTS memristor. These results suggest that the superior switching performance of the MTS memristor arises from the precise control of CFs through the use of the MTS structure, which adeptly governs the contact between the tenon-shaped top electrode and the HfO_2_ switching layer.

### The PDE solver based on the MTS memristors

**Fig. 4.**
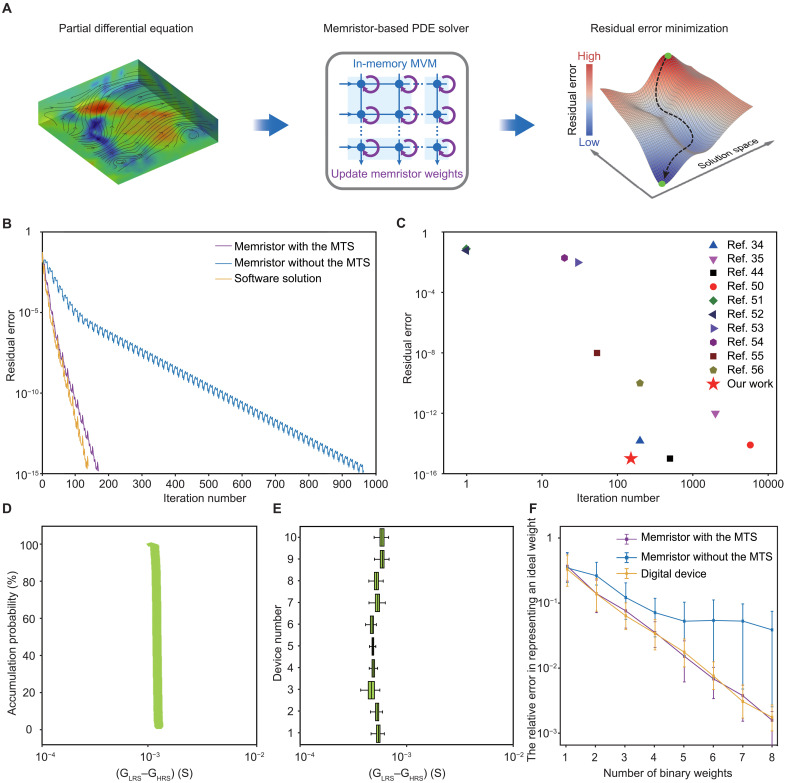
PDE solver based on the MTS memristor. (**A**) Schematic diagram illustrating the process of solving PDE in the in-memory PDE solver. The PDE solver minimizes the residual error of the equation and obtains accurate solutions by updating the memristor array weights and performing MVM operations. (**B**) Residual error convergence of solving the Poisson equation using the PDE solver based on the MTS memristors, HfO_2_ memristors, and software solution during flexible iterations. (**C**) Comparison of equation-solving efficiency (residual error versus iteration number) of different in-memory scientific computing schemes. (**D**) Cumulative probability plot of programmed weight represented by the difference in conductance between the LRS and the HRS (*G*_LRS_–*G*_HRS_) over 200 cycles of the MTS memristor. (**E**) The box-and-whisker plot shows the distribution of programmed weights represented by *G*_LRS_–*G*_HRS_ over 200 switching cycles for 10 MTS memristors. (**F**) The relationship between the relative error in representing an ideal weight using combined binary weights and the number of binary weights used. The combined weight is represented by memristors with the MTS structure, memristors without the MTS structure, and ideal digital devices.

By leveraging the high uniformity of the MTS memristors, we can achieve high-efficiency solutions for PDEs at a faster speed while using less hardware resources, compared to previous works (*34*, *35*, *44*, *50*–*56*). In Fig. 4A, we illustrate the application of the MTS memristors in solving a typical PDE, the Poisson equation describing the fluid behavior (*57*, *58*). In this approach, we use the MTS memristors to store the parameter matrix associated with the equation and use the memristor-based in-memory computing hardware to accelerate the MVM within the solution algorithm. Details about programming the parameter matrix in the memristors are provided in note SI. According to the flexible iteration algorithm, after each iteration of the calculation cycle, the memristor resistance is updated in real time (*59*–*61*). The flowchart of the algorithm is shown in fig. S12. This process gradually reduces the complexity of the equation solution and minimizes the difference between the solver output and the expected result by continuously minimizing the residual error, which improves the accuracy of the PDE solution (see more discussion about the solution process in note SII). In Fig. 4B, we evaluate the computing efficiency of the proposed solver using the MTS memristors compared to the solver using memristors without the MTS structure. The results show that the MTS memristor–based PDE solver reduces the residual error to 10^−15^ after 170 iterations. In contrast, the solver using the memristors without the MTS structure requires over 960 iterations to achieve equivalent performance. The use of the MTS memristors can improve the convergence speed of the solver by more than five times. Notably, the convergence speed of the MTS memristor–based PDE solver is comparable to that of software solutions (i.e., 150 iterations). Furthermore, we compare the solving efficiency of the MTS memristor–based PDE solver to other in-memory computing solver schemes (*34*, *35*, *44*, *50*–*56*), with the corresponding results shown in Fig. 4C. The results show that the MTS memristor–based PDE solver allows for the highest accuracy while reducing the number of iterations required. The higher solving efficiency of the PDE solver based on MTS memristors leads to considerable energy savings during the solution process. Under the condition of solving the same problem and achieving the same residual error convergence accuracy, we estimate the energy consumption of each of the three approaches: a solver based on high-uniformity MTS memristors, a solver based on conventional memristors, and a GPU-accelerated software (MATLAB) solver (using the 2020 NVIDIA A100 GPU, built on a 7-nm process). The results show that the energy consumption (18.1 mJ) of the MTS memristor–based solver is much lower than that of the conventional memristor (343.1 mJ) and the MATLAB software (769.9 mJ) (see note SIII and fig. S13).

The superior performance of the MTS memristors in the PDE solver can be attributed to their high uniformity, which notably enhances the accuracy of weight programming. In this way, the MTS memristors can be programmed to an LRS (or HRS) using a single set (or reset) voltage pulse with fixed parameters. The difference in programmed conductance between two MTS memristors was used to represent “1” or “0” binary values for the solver’s numerical weight (fig. S14). Accordingly, the uniformity of the programmed weight can be characterized as $C_{v-W}=\frac{\sqrt{C_{v-L}^{2}\times R^{2}+C_{v-H}^{2}}}{R-1}$, where *C*_v-L_ and *C*_v-H_ represent the uniformity of the LRS (~2.5%) and the HRS (~27.2%), respectively (as shown in Fig. 2C), and *R* denotes the on/off ratio (~10^2^) of the programmed resistive state. As shown in Fig. 4D, the minimum *C*_v-W_ of the MTS memristors is ~2.5%, which is the lowest value among reported HfO₂-based memristors so far (table S1). We further evaluate the programming weight uniformity across 10 devices, with the corresponding results shown in Fig. 4E and table S4. The results reveal that an average value *C*_v-W_ of about 9% can be achieved.

The improved uniformity of the programmed weights allows the solver to combine multiple binary weights for precise representation (note SIV and fig. S15). With this approach, when multiple MTS memristors are programmed and combined to represent weights, the relative error between the ideal weight and the combination of binary weights decreases as the number of binary weights increases, which is comparable to that based on the digital device (Fig. 4F). In contrast, the uniformity of traditional HfO_2_ memristors without the MTS structure is notably lower than that of the MTS memristors when programming weights (see fig. S16 and table S5).

## DISCUSSION

In summary, we propose a mortise-and-tenon h-BN/HfO_2_ memristor featuring a mortise-shaped h-BN flake and a tenon-shaped top electrode. This memristor exhibits the smallest cycle-to-cycle variation (~2.5%) and the smallest device-to-device variation (~6.9%). Through in situ C-AFM measurements, we reveal that the confinement of the switching region through the MTS structure allows for the reduction of the stochastic nature of conductive channel formation, thereby drastically improving the uniformity of devices. Furthermore, we demonstrate that the PDE solver using the MTS memristors exhibits a faster convergence speed than that based on traditional memristors. The proposed highly uniform MTS memristor offers a promising solution for implementing scientific computing more quickly and accurately while using less hardware resources.

## MATERIALS AND METHODS

### Device fabrication

The multilayer h-BN was obtained by using the mechanical exfoliation method on a 300-nm-thick SiO_2_ wafer, where commercial h-BN flakes were used as received. The mortise and square pattern of h-BN was defined by the standard electron beam lithography (EBL) method, followed by dry etching in a reactive ion beam (RIE) etching system using CHF_3_/O_2_ as the etching gas. To fabricate the MTS memristor, firstly, a Ti/Au/Pd bottom electrode (5/35/20-nm thickness, 2-μm width) on a 300-nm-thick SiO_2_/Si substrate was deposited through photolithography and electron beam deposition processes. A 3-nm-thick HfO_2_ switching layer was then deposited by atomic layer deposition, followed by the transfer of a few-layer h-BN as the functional layer. Subsequently, an independent mortise with a diameter of 100 nm was patterned using EBL and RIE. Last, the tenon-shaped Ta(80 nm)/Pd(40 nm)/Au(20 nm) top electrode (2-μm width) was integrated on the h-BN surface, completing the overall fabrication of the MTS memristor.

### Characterizations

#### Thickness and electrical measurements

The thickness of h-BN and HfO_2_ was identified by AFM. Current-voltage switching curves and resistance measurements were performed using an Agilent B1500A parameter analyzer. A waveform generator/fast measurement unit module was used to generate the voltage pulse and measure the response current simultaneously in this experiment.

#### In situ C-FAM experiments

The external voltage was applied to the bottom electrode, and the Cr/Pt-coated Si tip was set to 0 V, which enabled the connection of the h-BN/HfO_2_ with an amperemeter inside the AFM system (Bruker Multimode 8). The current mapping images obtained through C-AFM were acquired by scanning the surface in a constant voltage contact scanning mode.

#### TEM measurements

TEM samples were prepared using focused ion beam (FIB) from the specimen fabricated on the standard Si/SiO_2_ substrates. HAADF-STEM images were used to analyze the structure using a probe aberration-corrected STEM (Cs-STEM, FEI Cubed Themis Z, FEI, USA).

#### Implementation of flexible in-memory PDE solver

To demonstrate the contribution of the high uniformity of MTS memristors in facilitating advanced algorithms, we designed a flexible in-memory PDE solver and simulated its performance in solving the Poisson equation. The Poisson equation is formulated as $\nabla u=\frac{\partial^{2}u}{\partial x^{2}}+\frac{\partial^{2}u}{\partial y^{2}}=\text{sin}\left(\pi x\right)\text{cos}\left(\pi y\right)$, with a grid size of 40 × 40. Using the finite difference method, this can be expressed as a linear system of equations with a dimension of 1600. The solver implements a flexible iterative algorithm by using MTS memristors to compute the coefficient and preconditioner matrices in this system. Within the solver, combinations of devices in an 8 × 2 MTS memristor array are used to represent individual signed matrix elements with high precision. The corresponding optical and SEM images of the 8 × 2 MTS memristor array are shown in fig. S17. Consequently, the values of all matrix elements in the solver can be obtained through electrical measurements. On the basis of this setup, the solution of the Poisson equation using the flexible in-memory PDE solver was simulated in MATLAB. We applied the same solver framework and testing methodology to the HfO_2_ memristor (with the MTS structure or without the MTS structure) and obtained equation-solving results under identical conditions.

## Supplementary Materials

### This PDF file includes:

Supplementary Text Figs. S1 to S19 Tables S1 to S5 References
