# Supplementary material for: Mortise-tenon–shaped memristors for scientific computing
Source: Sci Adv. 2025 Apr 30;11(18):eadu3309. doi: 10.1126/sciadv.adu3309 (PMC12042887; doi:10.1126/sciadv.adu3309)
Supplement: Supplementary file 1 — Supplementary Text Figs. S1 to S19 Tables S1 to S5 References [file sciadv.adu3309_sm.pdf]

Science Advances
AAAS

Supplementary Materials for

Mortise-tenon–shaped memristors for scientific computing

Weiqi Dang *et al.*

Corresponding author: Chen Pan, chenpan@njust.edu.cn; Shi-Jun Liang, sjliang@nju.edu.cn; Feng Miao, miao@nju.edu.cn

Sci. Adv. **11**, eadu3309 (2025)
DOI: 10.1126/sciadv.adu3309

This PDF file includes:

Supplementary Text
Figs. S1 to S19
Tables S1 to S5
References

Supplementary Text

Supplementary Note I: The programming algorithm of the MTS memristor

All MTS memristor weights were experimentally programmed using the same electrical parameters without employing program-and-verify techniques. This approach minimizes the overhead of peripheral circuits and programming algorithms in the memristor array, which makes programming accuracy directly dependent on the uniformity of the memristors. During electrical measurements and the in-memory PDE solver, the set operation was performed using 1.5 V pulses with a width of 2 μs, while the reset operation was conducted using -1.7 V pulses with the same width. Under these conditions, MTS memristors were programmed with highly uniform binary weights.

Leveraging this superior uniformity, we adopted a widely used approach to represent binary conductance (G) using the differential conductance of memristors. Here, "G_{LRS}-G_{LRS}" and "G_{LRS}-G_{HRS}" were assigned to represent binary states "0" and "1," respectively, as shown in Fig. S14A. Subsequently, we combined eight binary conductance (G_{ij}^1 to G_{ij}^8) to express conductance $G_{ij}=2^{-1}\times G_{ij}^1+2^{-2}\times G_{ij}^2+\ldots+2^{-8}\times G_{ij}^8$, where 2^{-1}, 2^{-2} … 2^{-8} are coefficients corresponding to G_{ij}^1, G_{ij}^2 … G_{ij}^8, respectively. During computation, the combined conductance G_{ij} was used to represent numerical weights W_{ij}, as illustrated in Fig. S14B.

In Fig. S14C, we present the hardware implementation of this computational process. The system employs amplifiers with different gain factors (ranging from 2^{-1} to 2^{-8}) to amplify the input voltage signal V_{in}. The amplified signals were then applied to an array of binary unit conductance elements composed of memristors. Using Kirchhoff's and Ohm's laws, matrix operations can be implemented with this setup, as demonstrated in Fig. S14D.

Supplementary Note II: The iteration algorithm used within the in-memory PDE solver.

In the in-memory PDE solver, we utilize the flexible in-memory iteration algorithm shown below, which involves outer and inner iterations. Flexible in-memory iteration deploys an advanced numerical solution method called flexible iteration in the in-memory PDE solver. Compared to the static iterative methods employed by conventional solutions, flexible iterative methods speed up the solving process by using dynamic coefficient matrices (called a preconditioner) rather than static coefficient matrices, enabling the optimization of the residual error convergence during the solving process (Fig. S18). In hardware, the in-memory solver

implements the preconditioner by continuously updating the weights of the memristor, which places high demands on the uniformity of the device.

The process for flexible in-memory iteration is as follows. First, the problem to be solved is initialized within the solver, and based on the problem's dimensions, a series of flexible preconditioners is generated using the Algebraic Multigrid (AMG) method and static preconditioners. Each flexible preconditioner is employed during several inner iterations. After a number of inner iterations, the solver's flexible preconditioner is updated to further optimize the equation's convergence, and the current residual error and solution of the equation are updated. At the hardware level, MTS memristor arrays are used to store the coefficient matrix and preconditioner matrices and to perform matrix-vector multiplication operations.

Algorithm 1: Flexible In-memory Iteration

// To solve the equation $A\vec{x} = \vec{b}$

Input: A, $\vec{b}$, r_{target}

Output: $\vec{x}$

Initialize $\vec{x} = 0, \vec{r} = \vec{b}, i = 0$;

$[M_f^1, M_f^2 ... M_f^k] \leftarrow$ **Flexible AMG Preconditioner**(A, H);

for $\|\vec{r}\| > r_{target}$ **do**

  $\Delta\vec{x} \leftarrow$ **Inner FCG iteration**$(A, \vec{r}, s, M_f^i)$;

  $\vec{x} \leftarrow \vec{x} + \Delta\vec{x}$;

  $\vec{r} \leftarrow \vec{b} - A \cdot \vec{x}$;

  $i++$;

end

Specifically, we utilize the AMG method to construct the flexible preconditioner. The AMG method accelerates the convergence of residuals by gridifying the original equation and solving it at various coarser grid levels, effectively speeding up the convergence of low-frequency residual error components—a task that conventional iterative methods (such as stationary iteration methods) struggle with. Furthermore, since AMG preconditioning operates at the coarse grid level, the matrix operations involved are of smaller dimensions, significantly reducing the computational complexity of the preconditioning process.

In practical applications, we utilize an AMG method that uses a grid dimension coarsened by a factor of more than 4, yet still achieves high convergence efficiency, as shown in Fig. 4B. This indicates that the flexible in-memory iteration can effectively balance the trade-off between the computational complexity of preconditioning and its effectiveness. Additionally, we validated the performance of flexible preconditioners generated from various static preconditioners, including the Incomplete Cholesky (iChol) decomposition preconditioner (Fig. 4B), Gauss-Seidel preconditioner (Fig. S19A), and diagonal preconditioner (Fig. S19B). As demonstrated in Fig. S19,

the flexible in-memory iteration achieves convergence for the same problem across different static preconditioners and performs better than conventional preconditioned conjugate gradient (PCG) iteration methods that use only static preconditioners, with the solver's efficiency being correlated with the complexity of the static preconditioner used to generate flexible preconditioners. This suggests that the aforementioned method for constructing flexible preconditioners is both general and adaptable.

Algorithm 2: Flexible AMG Preconditioner

// Constructing a series of flexible preconditioner $[M_f^1, M_f^2 \ldots M_f^k]$ using the algebraic multigrid method based on a static preconditioner H

Input: A, H

Output: $[M_f^1, M_f^2 ... M_f^k]$

Initialize $[n_1, n_2 ... n_k]$;

// Generating a series of sparse grid dimensions $[n_1, n_2 \ldots n_k]$ based on the equation to be solved

$N \leftarrow$ **Rank**(A);

for $i < k$ **do**

$\quad M_f^i \leftarrow H + P_{N\times n_i}(R_{n_i\times N} \cdot A \cdot P_{N\times n_i})^{-1} R_{n_i\times N}(I - A \cdot H)$;

end

// $P_{N\times n_i}$ and $R_{n_i\times N}$ are transformation matrices between the fine grid N and the coarse grid n_i in the Algebraic Multigrid (AMG) method

We employed the flexible conjugate gradient (FCG) method as the algorithm for inner iterations. The FCG method is an advanced iterative algorithm widely used in scientific computing, known for its high convergence efficiency when solving symmetric positive definite linear systems. This makes it well suited for most scientific computation scenarios.

Algorithm 3: Inner FCG iteration

// Performing conjugate gradient iterations with a flexible preconditioner M_f

Input: A, $\vec{r}_0$, s, M_f

Output: $\vec{x}$

Initialize $\vec{x}_t$, $\vec{r}_t = \vec{r}_0$, $\vec{z}_t = M_f \cdot \vec{r}_0$, $\vec{p}_t = \vec{r}_0$ **for** $t = 1$ **to** s **do**

$\quad \alpha_t \leftarrow \frac{\vec{r}_t \cdot \vec{z}_t}{\vec{p}_t \cdot (A \cdot \vec{p}_t)}$;

$\quad \vec{x}_{t+1} \leftarrow \vec{x}_t + \alpha_t \cdot \vec{p}_t$;

$\quad \vec{r}_{t+1} \leftarrow \vec{r}_t - \alpha_t \cdot (A \cdot \vec{p}_t)$;

$\quad \vec{z}_{t+1} \leftarrow M_f \cdot \vec{r}_{t+1}$;

$\quad \beta_t \leftarrow \frac{\vec{r}_t \cdot \vec{z}_{t+1}}{\vec{r}_t \cdot \vec{z}_t}$;

$\quad \vec{p}_{t+1} \leftarrow \vec{z}_{t+1} + \beta_t \cdot \vec{p}_t$;

end

$r \leftarrow r_0 - A \cdot x_t$;

$x \leftarrow x_t$;

Supplementary Note III: Energy consumption estimation and comparison of PDE solvers based on memristors with the MTS structure, memristors without the MTS structure, and software (MATLAB) solution.

We estimated the total energy consumption for using three different schemes (a solver based on high-uniformity MTS memristors, a solver based on conventional memristors, and the MATLAB software) to solve the Poisson equation. The corresponding results are shown in Fig. S13. The energy consumption of the MTS memristor-based solver is 18.1 mJ, which corresponds to only 5.3% of the energy consumed by the conventional memristor-based solver (343.1 mJ) and 2.3% of the energy consumed by the MATLAB-based solver (769.9 mJ).

We used the following equation to estimate the energy consumption (E_{total}),

$$E_{\text{total}} = N_{\text{analogMVM}} \times E_{\text{analogMVM}} + N_{\text{programming}} \times E_{\text{programming}} + N_{\text{digitalMVM}} \times E_{\text{digitalMVM}} \quad \text{(S1)}$$

Here, $E_{\text{analogMVM}}$ represents the power consumption of implementing a single matrix-vector multiplication (MVM) operation with the memristor array. This value encompasses the energy expenditure of both the analog-to-digital conversions (ADCs) and digital-to-analog conversions (DACs). $N_{\text{analogMVM}}$ represents the number of MVM executions in the analog domain. $E_{\text{programming}}$ represents the energy required for a single programming event on either the MTS memristor array or the conventional memristor array. $N_{\text{programming}}$ refers to the corresponding number of programming operations. $E_{\text{digitalMVM}}$ refers to the energy consumed for a single MVM calculation executed by digital hardware (GPU). $N_{\text{digitalMVM}}$ corresponds to the total number of running MVM computations on the GPU.

$\boldsymbol{E}_{\textbf{analogMVM}}$ can be calculated based on the following equation:

$$E_{\text{analogMVM}} = S \times N^2 \times t_{\text{operation}} \times V_{\text{read}}^2 \times G_{\text{max}} + N_{\text{ADC}} \times E_{\text{ADC}} + N_{\text{DAC}} \times E_{\text{DAC}} \quad \text{(S2)}$$

Here, S represents the sparsity of the matrix utilized for MVM calculations performed by the memristor array. N is the total number of elements within the matrix. $t_{operation}$ is the time required for the MVM calculation. V_{read} is the read voltage of the memristor during the MVM calculation. G_{max} is the maximum conductance of the memristor weight. N_{ADC} is the number of ADCs required for the MVM calculation. E_{ADC} is the energy cost for each ADC. N_{DAC} is the number of DACs required for the MVM calculation. E_{DAC} is the energy cost for each DAC. Based on Eq. (S2), the energy consumption for each analog matrix-vector multiplication (MVM) operation within the PDE solver is calculated to be 1.9×10^{-5} J. For each sparse matrix MVM operation, the energy consumption is determined to be 2.4×10^{-6} J, as shown in Fig. S13B. Note that we have used the relevant parameters (i.e., $t_{operation}$, V_{read}, G_{max}) of the memristor and the energy consumption data

for ADCs (E_{ADC}) *(62)* and DACs (E_{ADC}) *(63)* reported in the literature to calculate the energy consumption.

$\boldsymbol{E_{programming}}$ can be calculated based on the following equation:

$$E_{\mathrm{programming}} = N_{\mathrm{array}} \times E_{\mathrm{array}} \quad \text{(S3)}$$

$$E_{\mathrm{array}} = N_{\mathrm{device}} \times t_{\mathrm{switch}} \times V_{\mathrm{set}} \times I_{\mathrm{set}} \quad \text{(S4)}$$

Here, N_{array} is the operation number of programming memristor array. E_{array} is the energy consumption of performing a programming operation on the memristor array. N_{device} is the total number of devices in the memristor array. E_{device} is the energy consumed to program a single device on the memristor array. t_{switch} is the time required for the programming of a single memristor. V_{set} is the set voltage of the memristor. I_{set} is the current flowing through the memristor during the programming operation (*64*).

Based on Eq. (S3-4), E_{array} is estimated to be 5.4×10^{-6} J for the array based on MTS memristors, and E_{array} is estimated to be 8.4×10^{-5} J for the array based on conventional memristors, as shown in Fig. S13B.

$\boldsymbol{E_{digitalMVM}}$ can be calculated based on the following equation:

$$E_{\mathrm{digitalMVM}} = N_{\mathrm{MAC}} \times E_{\mathrm{MAC}} \quad \text{(S5)}$$

Here, N_{MAC} is the number of the Multiply-accumulate (MAC) operations in each MVM calculation. E_{MAC} is the energy consumed by digital hardware (GPU) for each MAC operation. Based on Eq. (S5), the energy consumption for each MVM calculation in the digital domain is 5.1×10^{-3} J, and the energy consumption for each sparse matrix MVM calculation in the digital domain is 1.3×10^{-5} J, as shown in Fig. S13B. Note that the N_{MAC} for MVM calculation in the digital domain is 2.56×10^{6}, and the N_{MAC} for sparse matrix MVM calculation in the digital domain is 6.4×10^{3} for this specific computing task. We have used the 7-nm node NVIDIA A100 GPU (which has been widely used in industry), and the E_{MAC} is 2×10^{-9} J according to the literature. (*65-67*)

We have calculated the power consumption for a single execution of each of the six computational tasks involved in the solving process. Subsequently, we analyzed the frequency of task execution for each of the three computational schemes. The computational steps required to solve the same problem with identical residual error convergence accuracy were evaluated based

on the designed algorithm. For the memristor-based approach, each iteration performs 4 MVM operations and 4 sparse matrix MVM operations in the analog domain. Additionally, every 5 iterations include 4 extra sparse matrix MVM operations in the analog domain, 1 sparse matrix MVM operation in the digital domain, and 16 programming operations for the memristor array. For the solver utilizing MTS memristors, 170 iterations are needed, comprising 680 (=170×4) analog MVM operations, 816 (=170×4 + 170/5×4) analog sparse matrix MVM operations, 544 (=170/5×16) programming operations, and 34 digital sparse matrix MVM operations (170/5), as shown in Fig. S13C. In comparison, the solver based on conventional memristors requires 960 iterations, which include 3,840 (=960 × 4) analog MVM operations, 4608 (=960×4 + 960/5×4) analog sparse matrix MVM operations, 3072 (=960/5 × 16) programming operations, and 192 (=960/5) digital sparse matrix MVM operations, as shown in Fig. S13C. Besides, for the MATLAB-based approach, each iteration involves 1 matrix-vector multiplication (MVM) in the digital domain and 1 sparse matrix MVM in the digital domain, requiring a total of 150 iterations, as shown in Fig. S13C.

Consequently, the total energy consumption of the three schemes can be estimated as follows:

1) For the MTS memristor-based scheme:

$E_{\text{total}} = 680 \times 1.9 \times 10^{-5} + 816 \times 2.4 \times 10^{-6} + 544 \times 5.4 \times 10^{-6} + 34 \times 1.3 \times 10^{-5} = 18.1$ mJ

2) For the traditional memristor-based scheme:

$E_{\text{total}} = 3840 \times 1.9 \times 10^{-5} + 4608 \times 2.4 \times 10^{-6} + 3072 \times 8.4 \times 10^{-5} + 192 \times 1.3 \times 10^{-5} = 343.1$ mJ

3) For the MATLAB-based calculation scheme:

$E_{\text{total}} = 150 \times 5.1 \times 10^{-3} + 150 \times 1.3 \times 10^{-5} = 769.90$ mJ

Therefore, we conclude that our proposed solver based on the h-BN mortise memristors exhibits the smallest energy consumption.

Supplementary Note IV: Comparison of weight error between analog devices and digital devices

The ability to reduce weight error by representing a single weight with multiple devices is essential for achieving high-precision in-memory PDE solver. The digital in-memory computing scheme assigns one bit per device, and its accuracy scales with encoding specifications (such as INT8 or FP16) via the digital computing unit. While this greatly reduces the requirement for device uniformity, it results in increased circuit and energy overhead. In contrast, analog in-memory computing schemes, such as those based on memristors, achieve accuracy scaling by directly

assigning and accumulating electrical quantities in the analog domain. This approach places higher demands on device uniformity but is more energy efficient.

In the PDE solver, we use the differential conductance of two binary memristors to represent a binary conductance weight. For example, the conductance difference between a low-resistance state (LRS) and a high-resistance state (HRS) represents a weight of "1", while the conductance difference between two LRS devices represents a weight of "0" (see Supplementary Note I and Fig. S14). The higher uniformity of programmed weights enables the solver to achieve higher precision in weight representation by combining multiple binary weights. We accumulate the conductance of *n* binary weights in the simulation domain, multiplied by 2^{-1}, 2^{-2}, ..., 2^{-n}, to realize the combination of binary weights (see Fig. S15). The accuracy of weight representation in this context is determined by the uniformity of the memristors.

To assess the effect of memristor uniformity on weight representation accuracy, 800 weights uniformly distributed between 0 and 1 were generated. Using the proposed method, these weights were represented with *n* binary weights (*n* ranging from 1 to 8). A statistical analysis was performed to evaluate the error between the combined weights in the analog domain and the ideal weights, with the corresponding results presented in Fig. 4F. For *n*=8, memristors incorporating MTS achieved highly accurate weight quantification, with precision comparable to ideal 8-bit digital data. Conversely, the poor uniformity makes the memristors without the MTS fail to realize precision beyond 5 bits. These findings demonstrate that the MTS memristor featured with superior uniformity can extend the precision of weight representation in the analog domain, thereby simplifying solver design and drastically improving overall performance.

Supplementary Figures

I. The schematic diagram of the MTS fabrication process

II. The thickness of the HfO_2 switching layer

III. Detailed STEM images of the MTS memristor

IV. The distribution of V_{set}/V_{reset} for the MTS memristor

V. The resistive switching performance of the MTS memristor with thinner h-BN mortise.

VI. The switching speed measurement of the MTS memristor

VII. The resistive switching performance of the traditional HfO_2 memristor without the MTS structure.

VIII. The radar map of performance comparison between the MTS memristor and the traditional HfO_2 memristor

IX. The device-to-device uniformity of the 10 MTS memristors

X. The device-to-device uniformity of the 10 memristors without the MTS structure

XI. The device-to-device uniformity of 10 MTS memristors was evaluated by the box-and-whisker plot of LRS and HRS

XII. The flowchart of the algorithm used in the in-memory PDE solver

XIII. Energy consumption comparison of solvers based on memristors with and without the MTS structure, and the software (MATLAB) solution

XIV. The weight programming and representing scheme of the proposed in-memory PDE solver

XV. The construction of multi-bit weight based on the memristors

XVI. The uniformity of programmed weight for the traditional HfO_2 memristor without the MTS structure

XVII. The optical and SEM images of the 8×2 array

XVIII. Schematic diagram comparing the convergence effect of the flexible in-memory iteration method with the conventional solution

XIX. Comparison of the solution results of the flexible iterative method with conventional iterative methods using different static preconditioners

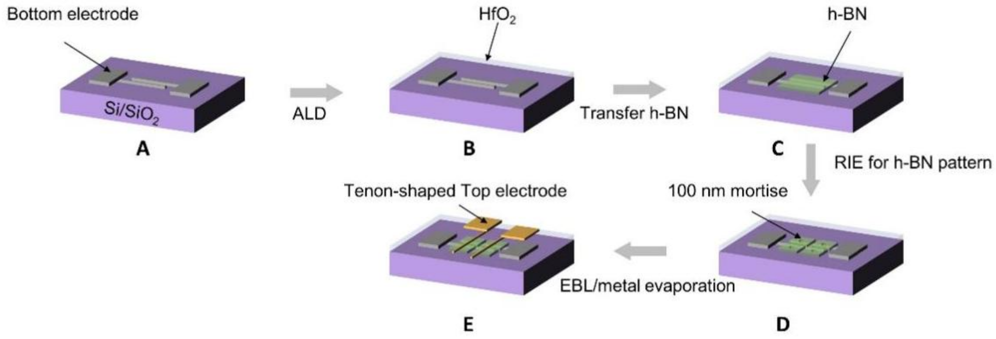

Fig. S1. The schematic diagram of the MTS memristor fabrication process. (**A**) Deposited Ti(5 nm)/Au(35 nm)/Pd(20 nm) on a standard SiO_2/Si substrate as the bottom electrode. **(B)** Deposited a 3 nm-thick HfO_2 switching layer by ALD, deposition rate: 7 Å/cycle. (**C**) Transferred the exfoliated h-BN nanosheet onto the channel area. (**D**) Defined the mortise of h-BN by EBL and RIE. h-BN pattern size: 5 μm×5 μm. (**E**) Integrated the top electrode Ta(80 nm)/Pd(40 nm)/Au(20 nm) onto the crossbar area.

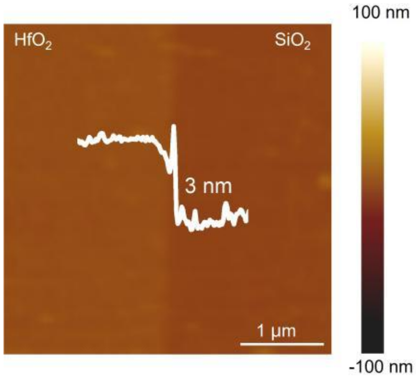

Fig. S2. The thickness of the HfO_2 switching layer. The test result of AFM shows that the thickness of HfO_2 is ~3 nm.

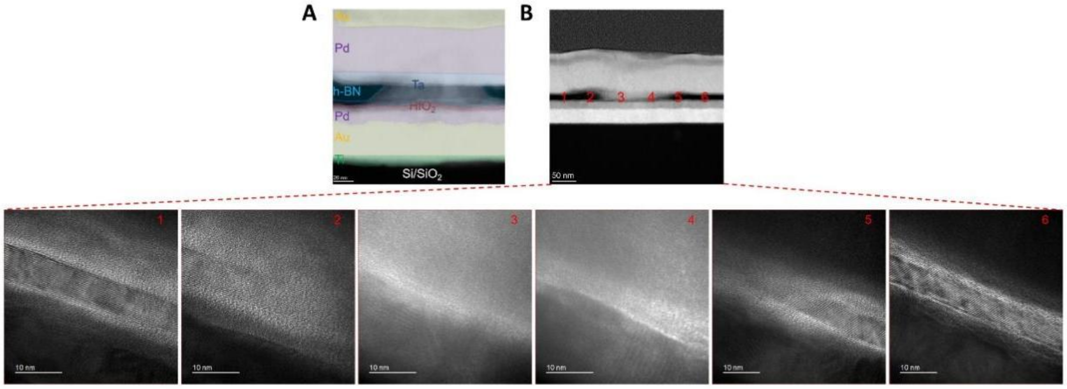

Fig. S3. Detailed STEM images of the MTS memristor. (**A**) The false-color STEM image shows a magnified view of the MTS memristor. (**B**) Cross-sectional HRTEM images of h-BN are displayed at various positions within the interface.

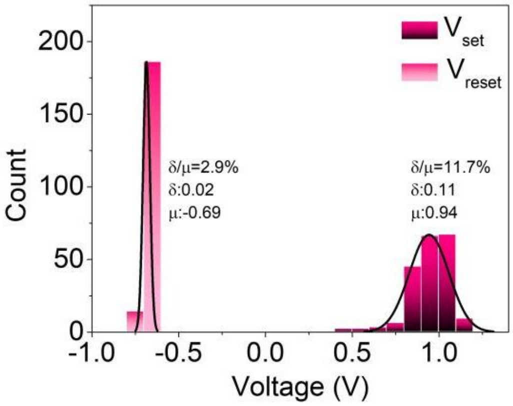

Fig. S4. The distribution of V_{set}/V_{reset} for the MTS memristor. 200 cycles of switching behavior of the MTS memristor, demonstrating low cycle-to-cycle variations. The C_v of the V_{set} and V_{reset} is 11.7% and 2.9%, respectively.

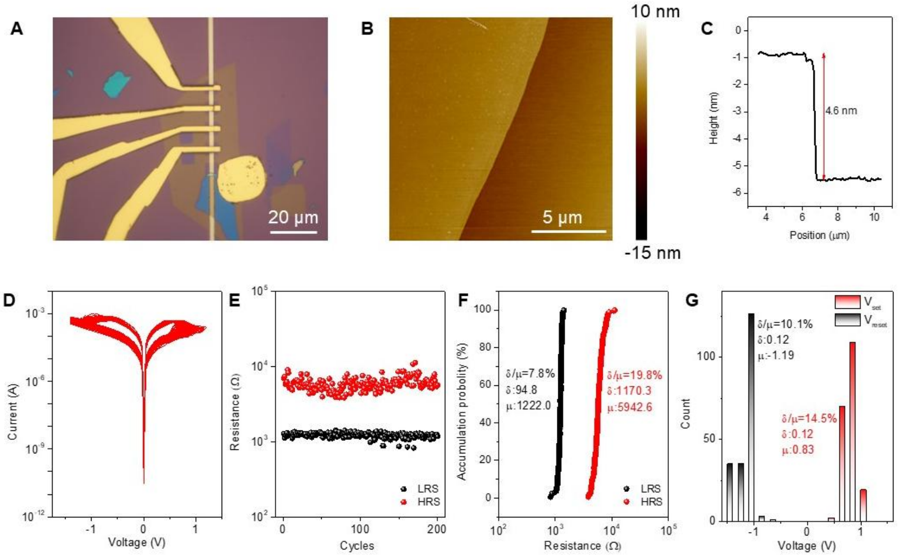

Fig. S5. The MTS memristor with ~4.6 nm h-BN. (**A**) The optical image of the MTS device with a thinner h-BN mortise, scale bar: 20 µm. (**B**) The original AFM image of h-BN in (A), scale bar: 5 µm. (**C**) The thickness of h-BN in (B), which is ~4.6 nm. (**D**) The *I-V* curve of the MTS device during 200 cycles in (A). (**E**) LRS/HRS data of device in (A). (**F**) The cumulative probability of the MTS device in (A), where the C_v of LRS is 7.8% and HRS is 19.8%, respectively. (**G**) The corresponding V_{set}/V_{reset} distribution of the MTS device in (A).

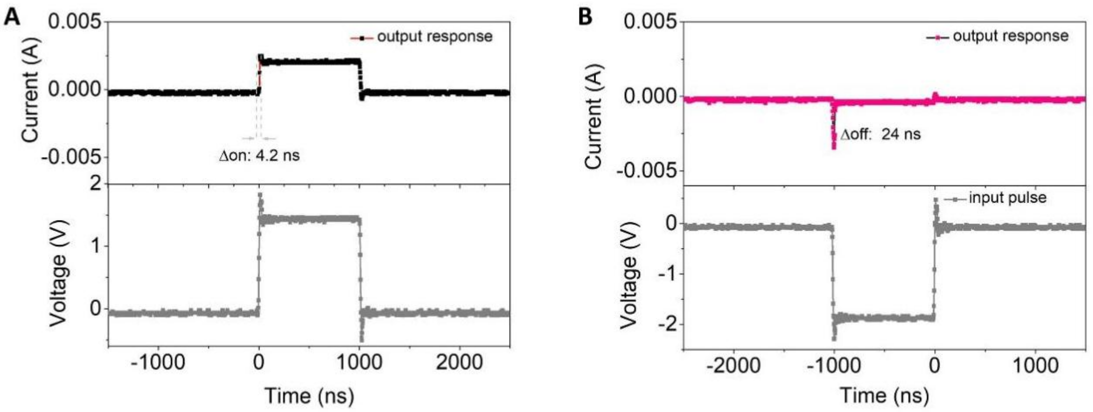

Fig. S6. The switching speed measurement of the MTS memristor. The programmed time in (A) and erased time in (B) of the MTS memristor. A fast switch-on time of 4.2 ns was measured, as shown in (A). This sub-10 ns switching speed illustrates the potential for sub-pJ power consumption in the MTS memristor.

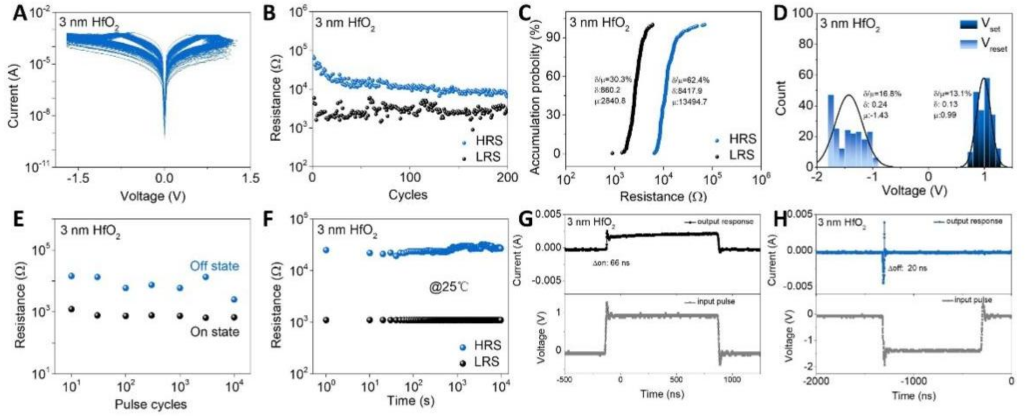

Fig. S7. The resistive switching performance of the traditional HfO_2 memristor without the MTS structure. (**A**) The *I-V* curve of the 3 nm-thick HfO_2 memristor without the MTS structure during 200 cycles. (**B**) The LRS/HRS data of the device in (A). (**C**) The cumulative probability of the 3 nm-thick HfO_2 memristor without the MTS structure, where the C_v of LRS is 30.3% and HRS is 62.4%, respectively. (**D**) The corresponding V_{set}/V_{reset} distribution of the device in (A). (**E**) The pulse endurance of the traditional memristor without the MTS structure (2 μs width with +1.9 V for SET and -1.9 V for RESET), with a limited endurance of 10^4 cycles. (**F**) The retention test of the device at room temperature, and an obvious HRS disturbance can be observed within 10^4 s. (**G, H**) The programming time in (G) and erase time in (H) of the traditional memristor without the MTS structure.

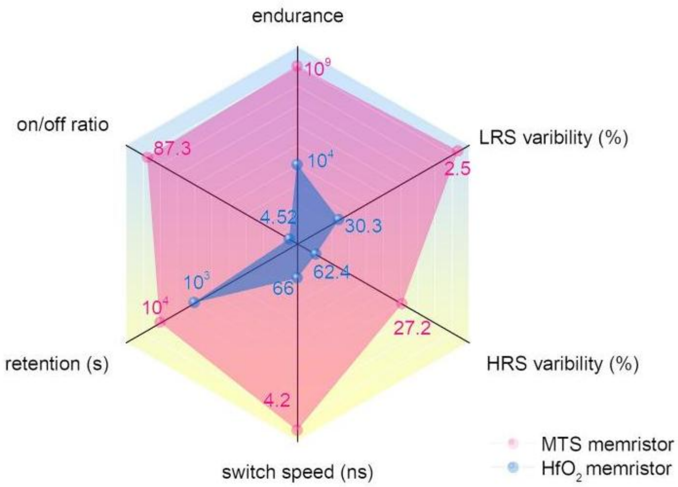

Fig. S8. The radar map of performance comparison between the MTS memristor and the traditional 3 nm-thick HfO_2 memristor. The results indicate that the uniformity, endurance, retention, and other performance parameters of the traditional 3 nm-thick HfO_2 memristor without the MTS structure were inferior to those of the stable MTS memristor, highlighting the crucial role of the MTS structure in enhancing HfO_2 memristor performance.

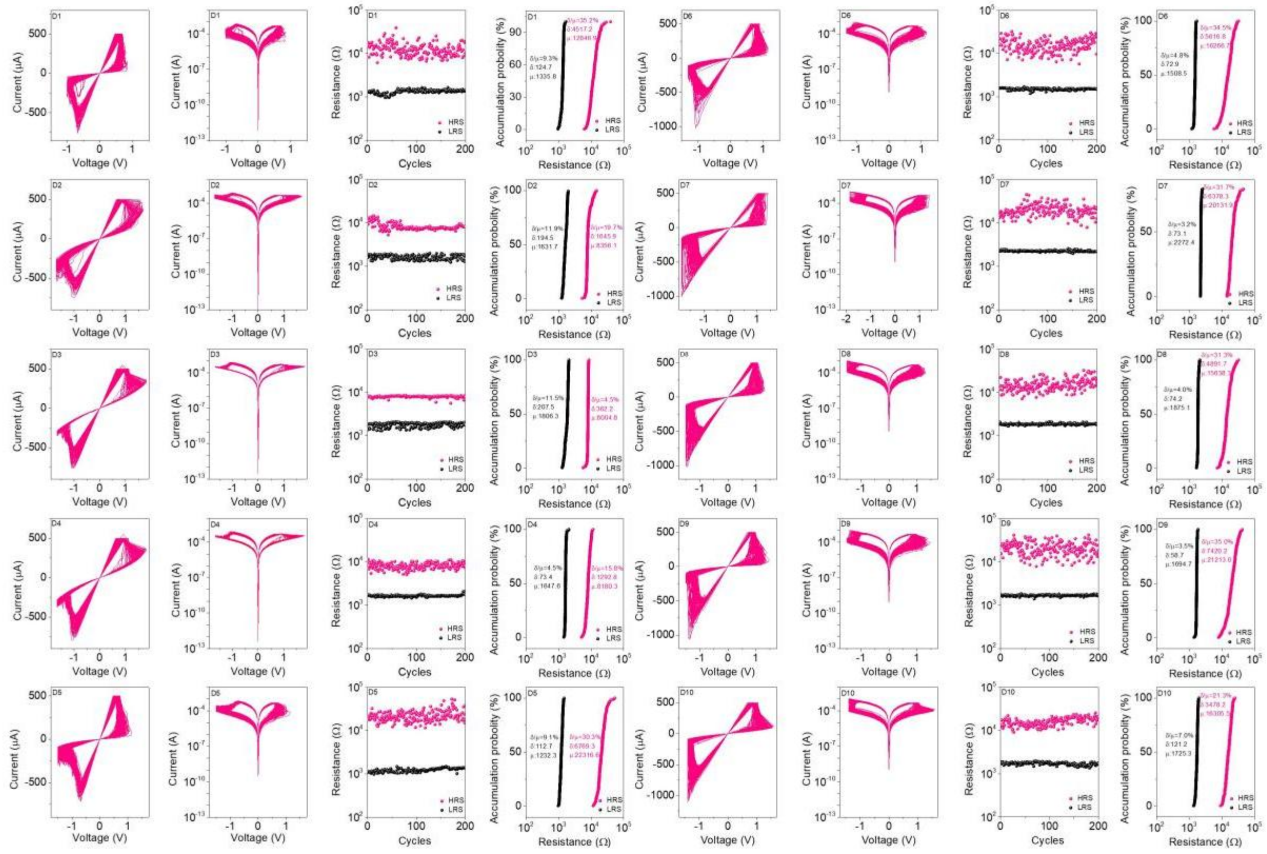

Fig. S9. The device-to-device uniformity of the 10 MTS memristors. The resistive switching performance of 10 MTS memristors characterizes the device-to-device uniformity. There are 10 sets of data corresponding to different devices, each set of data consists of 200 *I-V* curves (linear current coordinates and logarithmic current coordinates), LRS-HRS scatter plots, and LRS-HRS cumulative distribution curves.

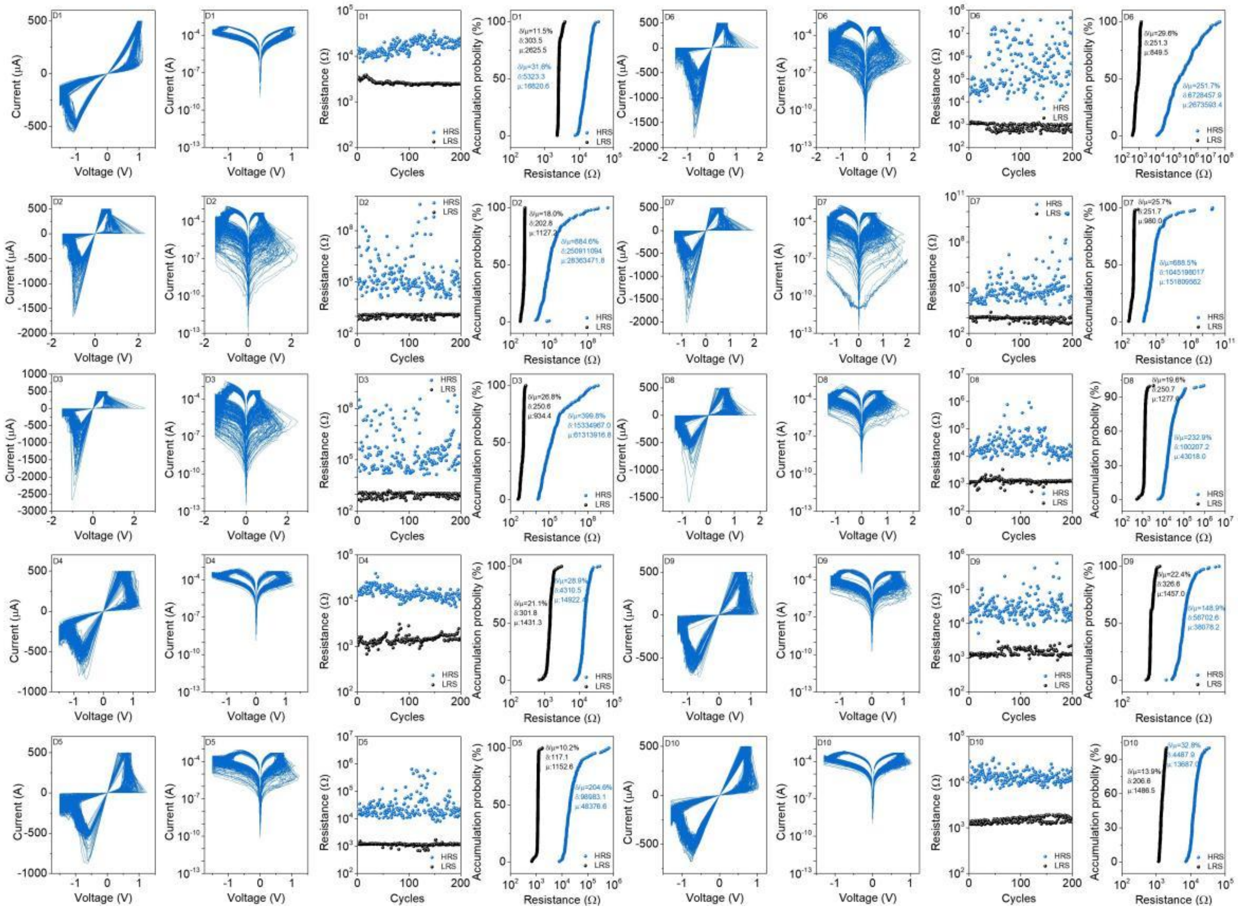

Fig. S10. The device-to-device uniformity of the 10 memristors without the MTS structure. The resistive switching performance of 10 memristors without the MTS structure characterizes the device-to-device uniformity. There are 10 sets of data corresponding to different devices, each set of data consists of 200 *I-V* curves (linear current coordinates and logarithmic current coordinates), LRS-HRS scatter plots, and LRS-HRS cumulative distribution curves.

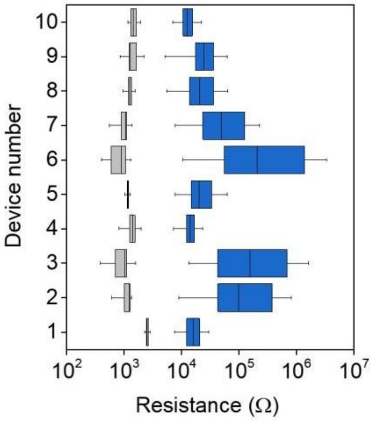

Fig. S11. The device-to-device uniformity of 10 MTS memristors was evaluated by the box-and-whisker plot of LRS and HRS. The distribution of both LRS and HRS fluctuates significantly over 200 cycles of switching behavior (the gray boxes and blue boxes represent LRS and HRS, respectively).

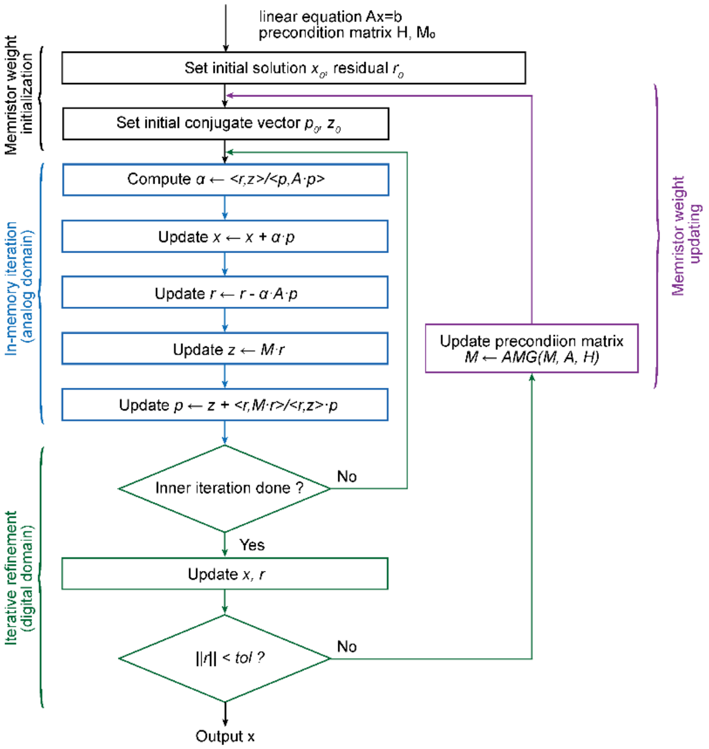

Fig. S12. The flowchart of the algorithm used in the in-memory PDE solver. The required computational steps include weight initialization, in-memory iteration, iterative refinement, and weight update steps.

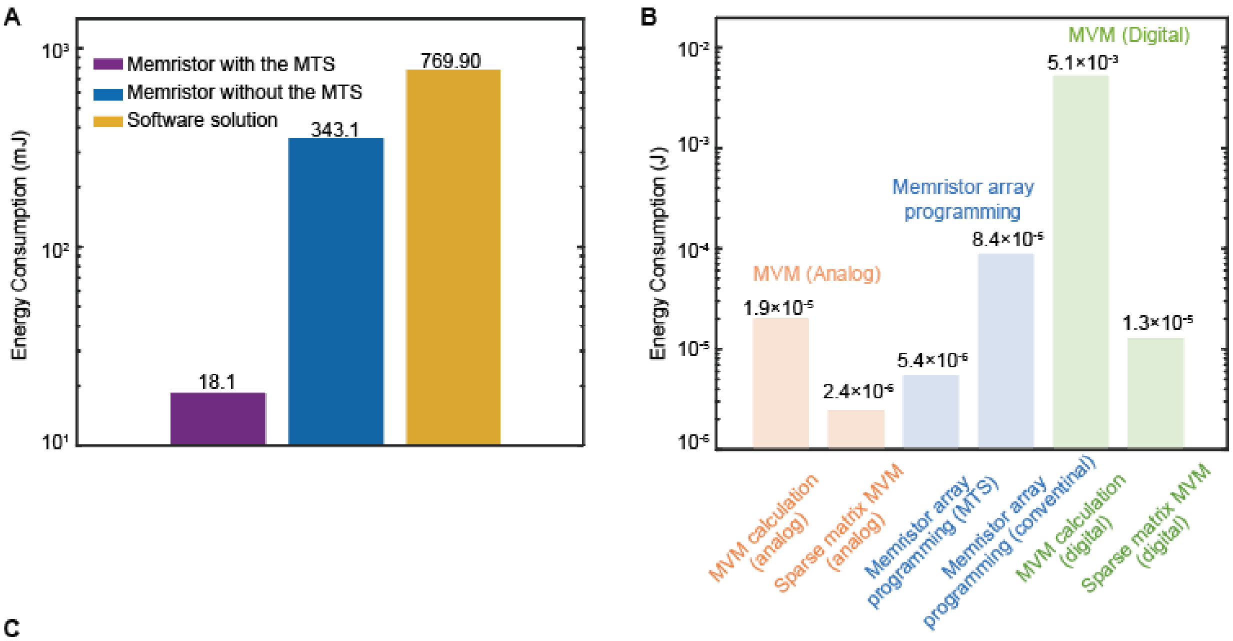

C

	MVM (Analog)		Memristor array programming		MVM (Digital)	
	MVM	Sparse matrix MVM	MTS memristor	Conventional memristor	MVM	Sparse matrix MVM
MTS Memristor-based Scheme	680	816	544	0	0	34
Conventional Memristor-based Scheme	3840	4608	0	3072	0	192
GPU-based MATLAB Scheme	0	0	0	0	150	150

Fig. S13. Energy consumption comparison of solvers based on memristors with and without the MTS structure, and the software (MATLAB) solution. (**A**) Comparison of total energy consumption across the three solutions. (**B**) Energy consumption for executing six basic operations performed by the PDE solver. (**C**) Number of various operations required to perform the equation solving until convergence under each solution.

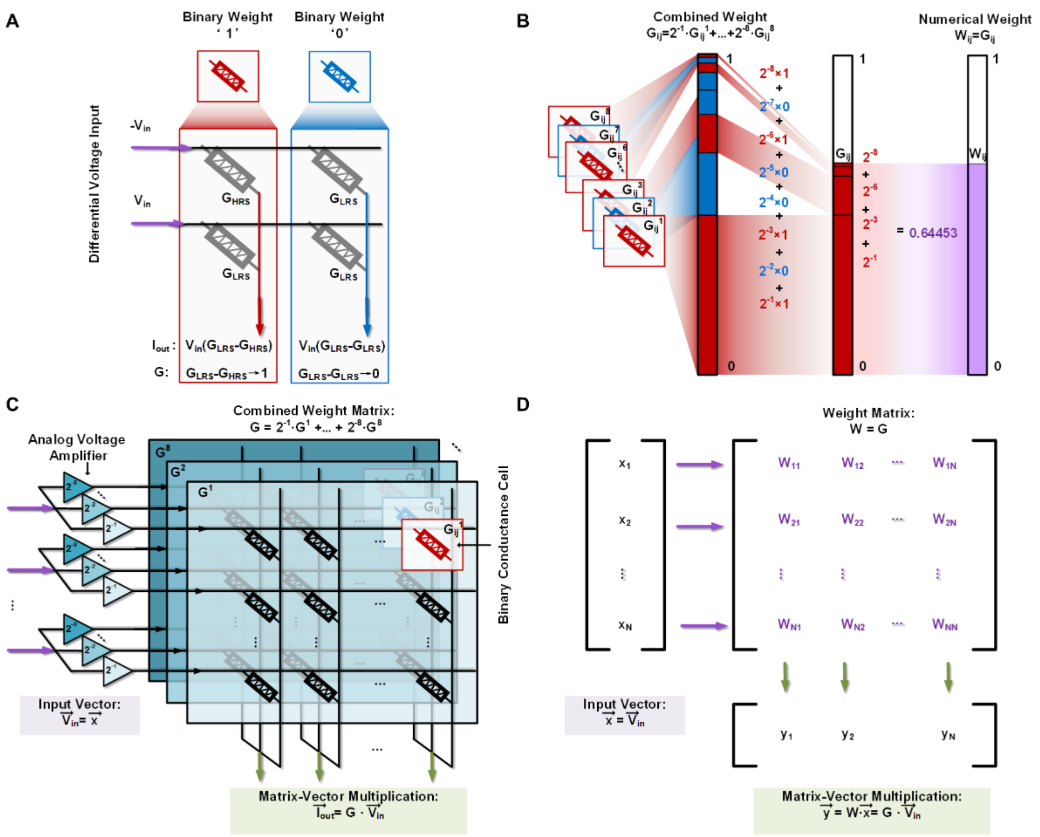

Fig. S14. The weight programming and representing scheme of the proposed in-memory PDE solver. (**A**) The schematic diagram of the scheme for representing weight "1" and "0" using the differential weight of two binarized memristors. By applying input voltages of V_{in} and -V_{in} to the two devices, the sum of the output current I_{out} is equivalent to the result of the differential input voltage V_{in} across the conductance of the two devices. (**B**) The schematic illustration of the programming method for representing high-precision numerical weight W_{ij} in the analog domain using a combination of binary conductance $G_{ij} = G_{ij}^1, G_{ij}^2, \ldots, G_{ij}^8$. (**C, D**) Schematic diagram of the circuit implementation of the weight representation scheme. The binary weight accumulation is achieved through the analog voltage gain, using an array of 8 binary memristor conductances to represent a matrix with high-precision numerical weights. Here, the input voltage V_{in} is equivalent to the vector $\vec{x}$ used in the matrix-vector multiplication (MVM) operation. The combined weight matrix G is equivalent to the numerical weight matrix W, and the output current $\overrightarrow{I_{out}}$ is equivalent to the MVM numerical computation result $\vec{y}$.

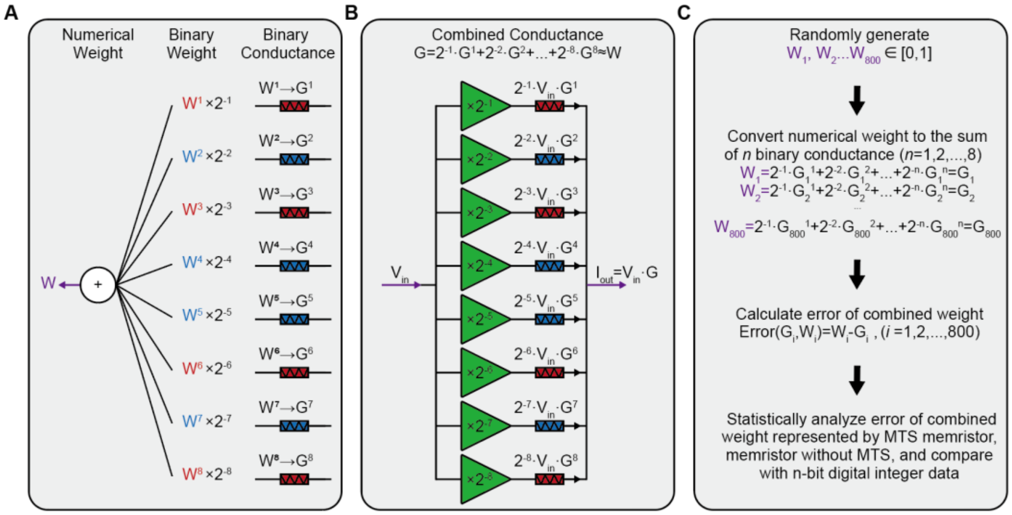

Fig. S15. The construction of multi-bit weight based on memristors. (**A**) Design scheme for constructing analog numerical weights using binary memristor combinations. (**B**) Hardware implementation circuit diagram of solution (A) using operational amplifiers and memristors. (**C**) For the circuit in Figure (B), the calculation method for the weight expression error is based on the nonuniformity of the device.

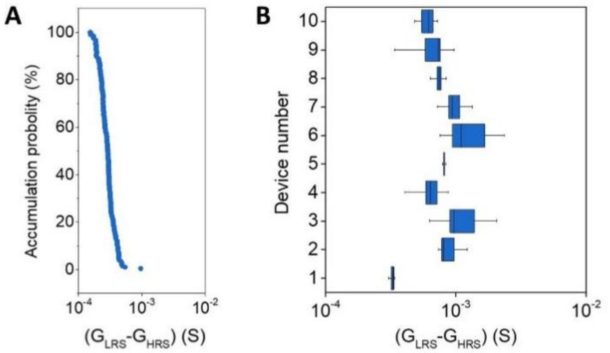

Fig. S16. The uniformity of programmed weight for the traditional HfO_2 memristor without the MTS structure. (**A**) Cumulative probability plots of programmed weight for the traditional HfO_2 memristor without the MTS structure after 200 cycles demonstrate an ultra-low variation of the programmed weight ($C_{V\text{-}W}$) of ~2.5%. (**B**) The device-to-device uniformity of 10 traditional HfO_2 memristors without the MTS structure was evaluated by the box-and-whisker plot of programmed weight, which displays the distribution of programmed weight after 200 cycles of switching behavior.

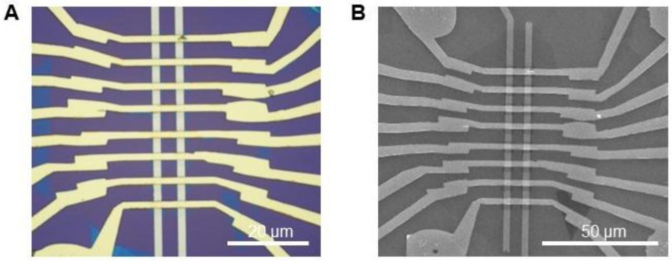

Fig. S17. The image of the MTS memristor array. (A) The optical image of the 8×2 array. The scale bar is 20 µm. **(B)** The corresponding SEM image of the 8×2 array. The scale bar is 50 µm.

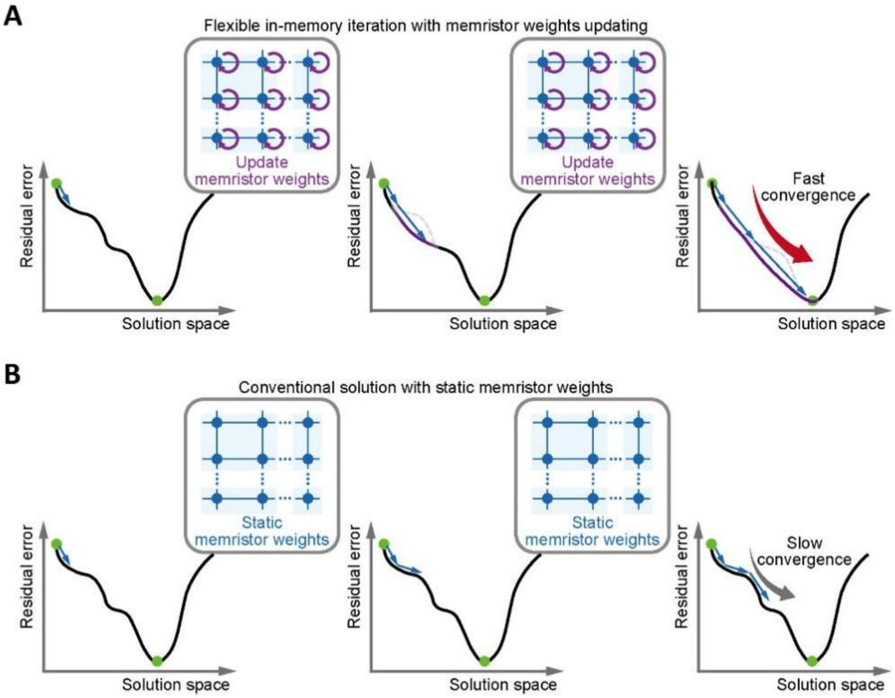

Fig. S18. Schematic diagram comparing the convergence effect of the flexible in-memory iteration method with the conventional solution. (**A**) Schematic of flexible in-memory iteration to optimize the convergence of residual error in solution space. By continuously updating the weights of the memristor, the local gradient of the residual error in the in-memory PDE solver increases, which leads to faster convergence. (**B**) Schematic of the conventional solution to optimize the convergence of residual error in solution space. Due to the lack of flexibility in the solver, the static memristor weights do not support the optimization of residual error convergence, which leads to slower convergence.

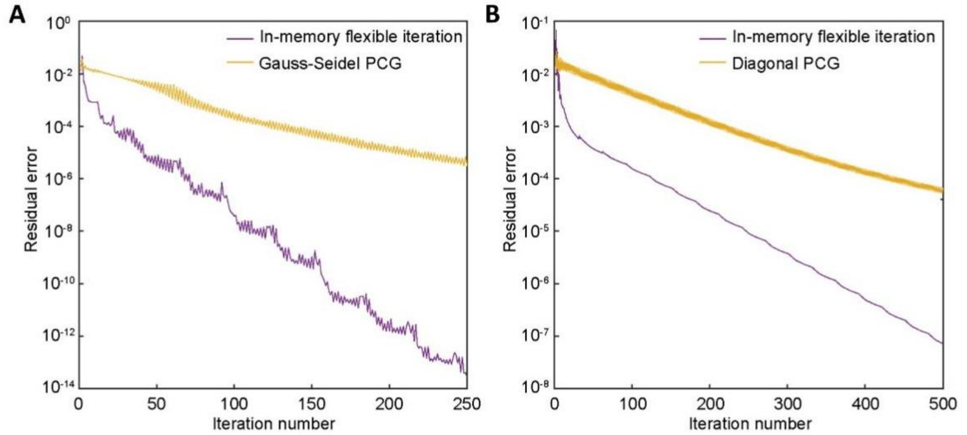

Fig. S19. Comparison of the solution results of the flexible iterative method with conventional iterative methods using different static preconditioners. (**A**) Residual error convergence of solving the Poisson equation using the in-memory flexible iteration method and the conventional PCG method with a static Gauss-Seidel preconditioner. (**B**) Residual error convergence of solving the Poisson equation using the in-memory flexible iteration method and the conventional PCG method with a static diagonal preconditioner.

Table S1. The resistive switching performance of HfO_2-based memristors for benchmark. (*1-25*)

structure	$V_{forming}$ (thickness)	DC cycles	V_{set} (C_v)	V_{reset} (C_v)	LRS (C_v)	HRS (C_v)	On/off ratio	$C_{v(LRS-HRS)}$	endurance	retention (@℃)	switch speed (ns)	ref
TiN/Ti/TiN/HfO_2/W	2-3 (5 nm)	N/A	0.5 (N/A)	-1 (N/A)	500 ohms (N/A)	10 kilohms (N/A)	10~100	19.3%	10^4	N/A	N/A	68
Ti/TiO_x/HfO_2/Pt	free-forming (9 nm)	100	0.88 (5.8%)	-0.89 (2.2%)	10 kilohms (N/A)	100 kilohms (N/A)	11.4	N/A	10^2	2×10^4 @RT	130	69
TiN/HfOx/graphene	free-forming	N/A	-0.2 (N/A)	0.2 (N/A)	100 kilohms (N/A)	10 megohms (N/A)	70	N/A	1.5×10^3	10^5 @RT	<500	70
Cu/HfO_2/Pt	3.7V (5 nm)	100	2 (~20%)	-3 (~50%)	1 kilohms (<20%)	100 megohms-10 gigaohms (>100%)	10^5~10^8	N/A	10^7	10^5@125	100	45
TiN/Hf/HfOx/TiN	5.3/2.3 (10 nm/5 nm)	100	0.5 (>100%)	-0.5 (<20%)	10 kilohms	1 megohm~0.1 gigaohms	>50	N/A	5×10^7	10^5@200	5	71
TiN/HfO_2/TiN	N/A (5 nm)	N/A	N/A	N/A	10 kilohms (N/A)	100 kilohms (N/A)	10	N/A	10^8	N/A	0.1	72
TiN/SLG/HfO_x/Pt	6.2 V (5 nm)	100	1.8 (N/A)	-3.2 (N/A)	3 megohms (>20%)	18 megohms (>100%)	6	N/A	N/A	10^5@100	N/A	73
ITO/MoS_2/Pd/HfO_x/ITO	N/A (15 nm)	N/A	-0.8 (N/A)	1 (N/A)	63 ohms (N/A)	0.1 megohms (N/A)	1600	N/A	2×10^2	10^4@85	N/A	74
TiN/HfO_x/TiN	N/A	N/A	1.4 (N/A)	-1.6 (N/A)	10 kilohms (N/A)	10 megohms (N/A)	10^3	N/A	10^{10}	N/A	0.3	75
TiN/TiO_x/HfO_x/TiN	2.1 (5 nm)	N/A	3.2 (N/A)	2.7 (N/A)	1 kilohms (N/A)	10 megohms (N/A)	10^4	N/A	$>10^6$	10 years@200	5	76
Pt/HfO_2NRs/TiN	free-forming (5~25 nm)	100	-2.5 (N/A)	1.5 (N/A)	10 kilohms 21.9%	1 megohms 31.2%	10^2	22.1%	10^2	10^4@85	N/A	77
Pt/HfO_2/Pt/HfO2/Ti	-5 V (10 nm)	100	0.95 (N/A)	-0.81 (N/A)	1.35 kilohms (5.3%)	0.92 megohms (11%)	6.8×10^2	5.3%	N/A	N/A	N/A	78
Pd/HfO_2/Ta	N/A (5 nm)	N/A	1.1 (N/A)	-1.3 (N/A)	2 kilohms (N/A)	2 megohms (N/A)	10^3	N/A	$>10^6$	10 yeras@ RT	50	79
ITO/HfOx/ITO	free-forming (5 nm)	400	0.63 4.3%	-2.88 4.3%	25 ohms (N/A)	1.125 kilohms (N/A)	45	N/A	5×10^7	$>10^6$	<20	80
Pt/HfO_2/Ta	2.02 (5 nm)	N/A	0.65 (N/A)	-1.1 (N/A)	0.5 kilohms (N/A)	17 kilohms (N/A)	34	N/A	1.2×10^{11}	>10 years at 85 °C	<5	81
TiN/HfO_2/HfO_2:Al/Pt	free-forming (8 nm)	100	0.8 (N/A)	-1.3 (N/A)	1 kilohms 6.4%	30 kilohms 22.4%	>10	7.5%	10^6	10^4 @85	N/A	82
TiN/HfO_x/AlO_y/Pt	5.1 (8 nm)	N/A	2.0 (N/A)	-2.0 (N/A)	1 kilohms (N/A)	12 kilohms (N/A)	>10	N/A	10^3	10^4@85	N/A	83
W/TiO_2/HfO_2/TaN	5.5 (10 nm)	N/A	1 (N/A)	-1.5 (N/A)	2 kilohms (N/A)	50 kilohms (N/A)	>10	N/A	$>10^7$	10^4@125	N/A	84
Pt/Al_2O_3/HfO_2/$HfAlO_x$/TiN	free-forming (12 nm)	1000	-1.3 (12.4%)	1.2 (1.87%)	0.1 kilohms (N/A)	12 kilohms (N/A)	10^2	N/A	600	10^4@RT	N/A	85
Pt/HfO_2/TiO_x/Ti	4.4 (15 nm)	100	1.75 (7.8%)	-1.5 (5%)	1 kilohms (N/A)	100 kilohms (N/A)	10^2	N/A	100	10^4@RT	N/A	86
TiN/Sb_2Te_3/HfO_2/Pt	7.5 (10 nm)	100	2.4 9.7%	-1.2 9.0%	200 ohms 13.7%	300 kilohms 30.9%	$>10^3$	130.7%	N/A	N/A	N/A	87
Pt/HfO_2/$BiFeO_3$/HfO_2/TiN	5.2 (12 nm)	100	1.25 7.0%	-0.9 3.1%	100 ohms (N/A)	1 megohms (N/A)	10^4	N/A	10^6	10^4@85	N/A	88
Ti/HfO_2/ITO/HfO_2/ITO/Ti	free-forming (5 nm)	100	0.6 14%	-0.65 5%	400 ohms (N/A)	10 kilohms (N/A)	>10	N/A	10^3	2×10^4@RT	N/A	89
Pt/GO/HfO_2/ITO	free-forming (20 nm)	100	2.2 4.5%	-3.1 5%	10 ohms (N/A)	10 kilohms (N/A)	10^3	N/A	5×10^8	N/A	N/A	90
Pt/HfO_2/Al_2O_3/TaN	-5.05 (5 nm)	N/A	-2.7 (N/A)	-1.9 (N/A)	300 ohms (N/A)	8 megohms (N/A)	$>10^3$	N/A	10^5	10^4@RT	270	91
**Ta/h-BN/HfO_2/Au**	**free-forming (3 nm)**	**200**	**0.64 (9.3%)**	**-0.69 (2.9%)**	**0.8 kilohms (2.5%)**	**19.9 kilohms (27.2%)**	**87**	**2.5%**	**10^9**	**10^4@RT**	**4.2**	**our work**

Table S2. C_v **of LRS and HRS of 10 MTS memristors and 10 memristors without the MTS structure**

	Memristor with MTS structure		Memristor without MTS structure	
	LRS	HRS	LRS	HRS
D1	9.3%	35.2%	11.5%	31.6%
D2	11.9%	19.7%	18%	884.5%
D3	11.5%	4.5%	26.8%	399.8%
D4	4.5%	15.8%	21.1%	28.9%
D5	9.1%	30.3%	10.2%	204.6%
D6	4.8%	34.7%	29.6%	251.7%
D7	3.2%	31.7%	25.7%	688.6%
D8	4.0%	31.3%	19.6%	232.9%
D9	3.5%	35.0%	22.4%	148.9%
D10	7%	21.3%	14.0%	32.8%

Table S3. C_v **of** V_{set} **and** V_{reset} **of 10 MTS memristors and 10 memristors without the MTS structure**

	Memristor with MTS structure		Memristor without MTS structure	
	V_{set}	V_{reset}	V_{set}	V_{reset}
D1	9.3%	7.3%	11.1%	5.1%
D2	14.7%	9.5%	37.1%	10.8%
D3	16.2%	5.3%	37.0%	11.7%
D4	16%	10.0%	12.0%	9.3%
D5	15.2%	13.5%	21.1%	13.6%
D6	12.6%	7.4%	27.8%	9.8%
D7	14.3%	12.0%	33.3%	7.6%
D8	12.9%	5.8%	23.9%	6.6%
D9	16.2%	7.7%	16.5%	5.7%
D10	16.5%	3.8%	11.5%	6.7%

Table S4. $C_{v\text{-}w}$ **of 10 MTS memristors**

	LRS	HRS	On/off ratio	$C_{v\text{-}w}$
D1	9.3%	35.2%	9.6	11.1%
D2	11.9%	19.7%	5.1	15.6%
D3	11.5%	4.5%	4.4	14.9%
D4	4.5%	15.8%	5.0	6.9%
D5	9.1%	30.3%	18.1	9.8%
D6	4.8%	34.7%	10.8	6.4%
D7	3.2%	31.7%	8.9	5.4%
D8	4%	31.3%	8.3	6.3%
D9	3.55%	35%	12.5	4.9%
D10	7%	21.3%	9.5	8.2%

Table S5. $C_{v\text{-}w}$ of 10 traditional HfO_2 memristors without the MTS structure

	LRS	HRS	On/off ratio	$C_{v\text{-}w}$
D1	11.5%	31.6%	6.4	14.8%
D2	18%	884.5%	24162.8	18.0%
D3	26.8%	399.8%	68618.5	26.8%
D4	21.1%	28.9%	10.4	23.5%
D5	10.2%	204.6%	42.0	11.6%
D6	29.6%	251.7%	3147.3	29.6%
D7	25.7%	688.6%	154907.7	25.7%
D8	19.6%	232.9%	33.7	21.4%
D9	22.4%	148.9%	26.1	24.0%
D10	14%	32.8%	9.2	16.2%

REFERENCES AND NOTES

1. G. Stefanou, The stochastic finite element method: Past, present and future. *Comput. Methods Appl. Mech. Eng.* **198**, 1031–1051 (2009).

2. H. Trac, U. L. Pen, A primer on eulerian computational fluid dynamics for astrophysics. *Publ. Astron. Soc. Pac.* **115**, 303–321 (2003).

3. R. Vacondio, C. Altomare, M. D. Leffe, X. Hu, D. L. Touzé, S. Lind, J.-C. Marongiu, S. Marrone, B. D. Rogers, A. Souto-Iglesias, Grand challenges for smoothed particle hydrodynamics numerical schemes. *Comput.. Part. Mech.* **8**, 575–588 (2021).

4. I. Boybat, M. L. Gallo, S. R. Nandakumar, T. Moraitis, T. Parnell, T. Tuma, B. Rajendran, Y. Leblebici, A. Sebastian, E. Eleftheriou, Neuromorphic computing with multi-memristive synapses. *Nat. Commun.* **9**, 2514 (2018).

5. C. Li, M. Hu, Y. Li, H. Jiang, N. Ge, E. Montgomery, J. Zhang, W. Song, N. Dávila, C. E. Graves, Z. Li, J. P. Strachan, P. Lin, Z. Wang, M. Barnell, Q. Wu, R. S. Williams, J. J. Yang, Q. Xia, Analogue signal and image processing with large memristor crossbars. *Nat. Electron.* **1**, 52–59 (2018).

6. A. Shafiee, A. Nag, N. Muralimanohar, R. Balasubramonian, J. P. Strachan, M. Hu, R. S. Williams, V. Srikumar, ISAAC: A convolutional neural network accelerator with in-situ analog arithmetic in crossbars, in *2016 ACM/IEEE 43rd Annual International Symposium on Computer Architecture (ISCA)* (2016).

7. P. M. Sheridan, F. Cai, C. Du, W. Ma, Z. Zhang, W. D. Lu, Sparse coding with memristor networks. *Nat. Nanotechnol.* **12**, 784–789 (2017).

8. C. Wang, S.-J. Liang, C.-Y. Wang, Z.-Z. Yang, Y. Ge, C. Pan, X. Shen, W. Wei, Y. Zhao, Z, Zhang, B. Cheng, C. Zhang, F. Miao, Scalable massively parallel computing using continuous-time data representation in nanoscale crossbar array. *Nat. Nanotechnol.* **16**, 1079–1085 (2021).

9. Z. Wang, S. Joshi, S. Savel'ev, W. Song, R. Midya, Y. Li, M. Rao, P. Yan, S. Asapu, Y. Zhuo, H. Jiang, P. Lin, C. Li, J. H. Yoon, N. K. Upadhyay, J. Zhang, M. Hu, J. P. Strachan, M.

Barnell, Q. Wu, H. Wu, R. S. Williams, Q. Xia, J. J. Yang, Fully memristive neural networks for pattern classification with unsupervised learning. *Nat. Electron.* **1**, 137–145 (2018).

10. W. Zhang, B. Gao, J. Tang, P. Yao, S. Yu, M.-F. Chang, H.-J. Yoo, H. Qian, H. Wu, Neuro-inspired computing chips. *Nat. Electron.* **3**, 371–382 (2020).

11. M. A. Zidan, J. P. Strachan, W. D. Lu, The future of electronics based on memristive systems. *Nat. Electron.* **1**, 22–29 (2018).

12. J. J. Yang, D. B. Strukov, D. R. Stewart, Memristive devices for computing. *Nat. Nanotechnol.* **8**, 13–24 (2013).

13. F. Aguirre, A. Sebastian, M. L. Gallo, W. Song, T. Wang, J. J. Yang, W. Lu, M.-F. Chang, D. Ielmini, Y. Yang, A. Mehonic, A. Kenyon, M. A. Villena, J. B. Roldán, Y. Wu, H.-H. Hsu, N. Raghavan, J. Suñé, E. Miranda, A. Eltawil, G. Setti, K. Smagulova, K. N. Salama, O. Krestinskaya, X. Yan, K.-W. Ang, S. Jain, S. Li, O. Alharbi, S. Pazos, M. Lanza, Hardware implementation of memristor-based artificial neural networks. *Nat. Commun.* **15**, 1974 (2024).

14. W.-H. Chen, C. Dou, K.-X. Li, W.-Y. Lin, P.-Y. Li, J.-H. Huang, J.-H. Wang, W.-C. Wei, C.-X. Xue, Y.-C. Chiu, Y.-C. King, C.-J. Lin, R.-S. Liu, C.-C. Hsieh, K.-T. Tang, J. J. Yang, M.-S. Ho, M.-F. Chang, CMOS-integrated memristive non-volatile computing-in-memory for AI edge processors. *Nat. Electron.* **2**, 420–428 (2019).

15. Y. Huang, T. Ando, A. Sebastian, M.-F. Chang, J. J. Yang, Q. Xia, Memristor-based hardware accelerators for artificial intelligence. *Nat. Rev. Electr. Eng.* **1**, 286–299 (2024).

16. D. Ielmini, H.-S. P. Wong, In-memory computing with resistive switching devices. *Nat. Electron.* **1**, 333–343 (2018).

17. M. Lanza, A. Sebastian, W. D. Lu, M. L. Gallo, M.-F. Chang, D. Akinwande, F. M. Puglisi, H. N. Alshareef, M. Liu, J. B. Roldan, Memristive technologies for data storage, computation, encryption, and radio-frequency communication. *Science* **376**, eabj9979 (2022).

18. A. Sebastian, M. Le Gallo, R. Khaddam-Aljameh, E. Eleftheriou, Memory devices and applications for in-memory computing. *Nat. Nanotechnol.* **15**, 529–544 (2020).

19. C. Wang, G.-J. Ruan, Z.-Z. Yang, X.-J. Yangdong, Y. Li, L. Wu, Y. Ge, Y. Zhao, C. Pan, W. Wei, L.-B. Wang, B. Cheng, Z. Zhang, C. Zhang, S.-J. Liang, F. Miao, Parallel in-memory wireless computing. *Nat. Electron.* **6**, 381–389 (2023).

20. Z. Wang, C. Li, W. Song, M. Rao, D. Belkin, Y. Li, P. Yan, H. Jiang, P. Lin, M. Hu, J. P. Strachan, N. Ge, M. Barnell, Q. Wu, A. G. Barto, Q. Qiu, R. S. Williams, Q. Xia, J. J. Yang, Reinforcement learning with analogue memristor arrays. *Nat. Electron.* **2**, 115–124 (2019).

21. C.-X. Xue, Y.-C. Chiu, T.-W. Liu, T.-Y. Huang, J.-S. Liu, T.-W. Chang, H.-Y. Kao, J.-H. Wang, S.-Y. Wei, C.-Y. Lee, S.-P. Huang, J.-M. Hung, S.-H. Teng, W.-C. Wei, Y.-R. Chen, T.-H. Hsu, Y.-K. Chen, Y.-C. Lo, T.-H. Wen, C.-C. Lo, R.-S. Liu, C.-C. Hsieh, K.-T. Tang, M.-S. Ho, M.-F. Chang, A CMOS-integrated compute-in-memory macro based on resistive random-access memory for AI edge devices. *Nat. Electron.* **4**, 81–90 (2021).

22. D. B. Strukov, G. S. Snider, D. R. Stewart, R. S. Williams, The missing memristor found. *Nature* **453**, 80–83 (2008).

23. M. Hu, C. E. Graves, C. Li, Y. Li, N. Ge, E. Montgomery, N. Davila, H. Jiang, R. S. Williams, J. J. Yang, Q. Xia, J. P. Strachan, Memristor-based analog computation and neural network classification with a dot product engine. *Adv. Mater.* **30**, 1705914 (2018).

24. L. Sun, Y. Zhang, G. Han, G. Hwang, J. Jiang, B. Joo, K. Watanabe, T. Taniguchi, Y.-M. Kim, W. J. Yu, B.-S. Kong, R. Zhao, H. Yang, Self-selective van der Waals heterostructures for large scale memory array. *Nat. Commun.* **10**, 3161 (2019).

25. T. Li, H. Yu, S. H. Y. Chen, Y. Zhou, S.-T. Han, The strategies of filament control for improving the resistive switching performance. *J. Mater. Chem. C* **8**, 16295–16317 (2020).

26. H. Kim, M. R. Mahmoodi, H. Nili, D. B. Strukov, 4K-memristor analog-grade passive crossbar circuit. *Nat. Commun.* **12**, 5198 (2021).

27. H. Li, S. Wang, X. Zhang, W. Wang, R. Yang, Z. Sun, W. Feng, P. Lin, Z. Wang, L. Sun, Y. Yao, Memristive crossbar arrays for storage and computing applications. *Adv. Intell. Syst.* **3**, 2100017 (2021).

28. J. Yang, F. Zhang, H.-M. Xiao, Z.-P. Wang, P. Xie, Z. Feng, J. Wang, J. Mao, Y. Zhou, S.-T. Han, A perovskite memristor with large dynamic space for analog-encoded image recognition. *ACS Nano* **16**, 21324–21333 (2022).

29. H.-X. Li, Q.-X. Li, F.-Z. Li, J.-P. Liu, G.-D. Gong, Y.-Q. Zhang, Y.-B. Leng, T. Sun, Y. Zhou, S.-T. Han, Ni single-atoms based memristors with ultrafast speed and ultralong data retention. *Adv. Mater.* **36**, 2308153 (2024).

30. Z. Wang, H. Wu, G. W. Burr, C. S. Hwang, K. L. Wang, Q. Xia, J. J. Yang, Resistive switching materials for information processing. *Nat. Rev. Mater.* **5**, 173–195 (2020).

31. X. Zhao, J. Ma, X. Xiao, Q. Liu, L. Shao, D. Chen, S. Liu, J. Niu, X. Zhang, Y. Wang, R. Cao, W. Wang, Z. Di, H. Lv, S. Long, M. Liu, Breaking the current-retention dilemma in cation-based resistive switching devices utilizing graphene with controlled defects. *Adv. Mater.* **30**, 1705193 (2018).

32. J. Lee, C. Du, K. Sun, E. Kioupakis, W. D. Lu, Tuning ionict transport in memristive devices by graphene with engineered nanopores. *ACS Nano* **10**, 3571–3579 (2016).

33. B. Feinberg, U. K. R. Vengalam, N. Whitehair, S. Wang, E. Ipek, Enabling scientific computing on memristive accelerators, in *Proceedings–International Symposium on Computer Architecture*). Institute of Electrical and Electronics Engineers Inc. (IEEE, 2018).

34. M. L. Gallo, A. Sebastian, R. Mathis, M. Manica, H. Giefers, T. Tuma, C. Bekas, A. Curioni, E. Eleftheriou, Mixed-precision in-memory computing. *Nat. Electron.* **1**, 246–253 (2018).

35. M. A. Zidan, Y. Jeong, J. Lee, B. Chen, S. Huang, M. J. Kushner, W. D. Lu, A general memristor-based partial differential equation solver. *Nat. Electron.* **1**, 411–420 (2018).

36. J. Li, S.-G. Ren, Y. Li, L. Yang, Y. Yu, R. Ni, H. Zhou, H. Bao, Y. He, J. Chen, H. Jia, X. Miao, Sparse matrix multiplication in a record-low power self-rectifying memristor array for scientific computing. *Sci. Adv.* **9**, eadf7474 (2023).

37. Q. Chen, Y. Zhou, W. Xiong, Z. Chen, Y. Wang, X. Miao, Y. He, Complementary memtransistors for neuromorphic computing: How, what and why. *J. Semicond.* **45**, 061701 (2024).

38. Y. Deng, Y. Li, P. Wang, S. Wang, X. Pan, D. Wang, Observation of resistive switching in a graphite/hexagonal boron nitride/graphite heterostructure memristor. *J. Semicond.* **43**, 052003 (2022).

39. X. Du, S. Wang, Q. Zhang, S. Chen, F. Yang, Z. Liu, Z. Fan, L. Ma, L. Wang, L. Du, Z. Wang, C. Wang, B. Chen, Q. Liu, Memristive feature and mechanism induced by laser-doping in defect-free 2D semiconductor materials. *J. Semicond.* **45**, 072701 (2024).

40. G. Pedretti, P. Mannocci, C. Li, Z. Sun, J. P. Strachan, D. Ielmini, Redundancy and analog slicing for precise in-memory machine learning-Part I: Programming techniques. *IEEE Trans. Electron Devices* **68**, 4373–4378 (2021).

41. B. Gao, Y. Zhou, Q. Zhang, S. Zhang, P. Yao, Y. Xi, Q. Liu, M. Zhao, W. Zhang, Z. Liu, X. Li, J. Tang, H. Qian, H. Wu, Memristor-based analogue computing for brain-inspired sound localization with in situ training. *Nat. Commun.* **13**, 2026 (2022).

42. Z. Jiang, Y. Xi, J. Tang, Y. Lu, R. Yu, R. Hu, B. Gao, H. Qian, H. Wu, COPS: An efficient and reliability-enhanced programming scheme for analog RRAM and on-chip implementation of denoising diffusion probabilistic Model, in *2023 International Electron Devices Meeting (IEDM)* (2023) pp. 1–4.

43. M. Rao, H. Tang, J. Wu, W. Song, M. Zhang, W. Yin, Y. Zhuo, F. Kiani, B. Chen, X. Jiang, H. Liu, H.-Y. Chen, R. Midya, F. Ye, H. Jiang, Z. Wang, M. Wu, M. Hu, H. Wang, Q. Xia, N. Ge, J. Li, J. J. Yang, Thousands of conductance levels in memristors integrated on CMOS. *Nature* **615**, 823–829 (2023).

44. W. Song, M. Rao, Y. Li, C. Li, Y. Zhuo, F. Cai, M. Wu, W. Yin, Z. Li, Q. Wei, S. Lee, H. Zhu, L. Gong, M. Barnell, Q. Wu, P. A. Beerel, M. S.-W. Chen, N. Ge, M. Hu, Q. Xia, J. J. Yang, Programming memristor arrays with arbitrarily high precision for analog computing. *Science* **383**, 903–910 (2024).

45. X. Zhao, S. Liu, J. Niu, L. Liao, Q. Liu, X. Xiao, H. Lv, S. Long, W. Banerjee, W. Li, S. Si, M. Liu, Confining cation iinjection to enhance CBRAM performance by nanopore graphene layer. *Small* **13**, 1603948 (2017).

46. M. Song, S. Lee, S. S. T. Nibhanupudi, J. V. Singh, M. Disiena, C. J. Luth, S. Wu, M. J. Coupin, J. H. Warner, S. K. Banerjee, Self-compliant threshold switching devices with high on/off ratio by control of quantized conductance in Ag filaments. *Nano Lett.* **23**, 2952–2957 (2023).

47. L. Zhao, Z. Jiang, H.-Y. Chen, J. Sohn, K. Okabe, B. Magyari-Köpe, H.-S. P. Wong, Y. Nishi, Ultrathin (□2nm) HfO_x as the fundamental resistive switching element: Thickness scaling limit, stack engineering and 3D integration, in *2014 IEEE International Electron Devices Meeting (IEDM)* (IEEE, 2014).

48. W. Tong, W. Wei, X. Zhang, S. Ding, Z. Lu, L. Liu, W. Li, C. Pan, L. Kong, Y. Wang, M. Zhu, S.-J. Liang, F. Miao, Y. Liu, Highly stable HfO_2 memristors through van der Waals electrode lamination and delamination. *Nano Lett.* **23**, 9928–9935 (2023).

49. X.-D. Li, N.-K. Chen, B.-Q. Wang, M. Niu, M. Xu, X. Miao, X.-B. Li, Resistive memory devices at the thinnest limit: Progress and challenges. *Adv. Mater.* **36**, 2307951 (2024).

50. H. Yang, P. Huang, Z. Zhou, Y. Zhang, R. Han, X. Liu, J. Kang, Mixed-precision partial differential equation solver design based on nonvolatile memory. *IEEE Trans. Electron Devices* **69**, 3708–3715 (2022).

51. Z. Sun, G. Pedretti, E. Ambrosi, A. Bricalli, W. Wang, D. Ielmini, Solving matrix equations in one step with cross-point resistive arrays. *Proc. Natl. Acad. Sci. U.S.A.* **116**, 4123–4128 (2019).

52. P. Mannocci, E. Melacarne, A. Pezzoli, G. Pedretti, C. Villa, F. Sancandi, U. Spagnolini, U. Spagnolini, An SRAM-based reconfigurable analog in-memory computing circuit for solving linear algebra problems, in *2023 International Electron Devices Meeting (IEDM)* (2023) pp. 1–4.

53. J. Li, H. Zhou, Y. Li, X. Miao, A memristive neural network based matrix equation solver with high versatility and high energy efficiency. *Sci. China Inf. Sci.* **66**, 122402 (2022).

54. S. S. Ensan, S. Ghosh, ReLOPE: Resistive RAM-based linear first-order partial differential equation solver. *IEEE Trans. Very Large Scale Integr. VLSI Syst.* **29**, 237–241 (2021).

55. *L. Song*, F. Chen, H. Li, Y. Chen, ReFloat: Low-cost floating-point processing in ReRAM for accelerating iterative linear solvers, in *Proceedings of the International Conference for High Performance Computing*, *Networking*, *Storage and Analysis*, (2023) pp. 1–15.

56. B. Feinberg, R. Wong, T. P, Xiao, C. H. Bennett, J. N. Rohan, E. G. Boman, M. J. Marinella, S. Agarwal, E. Ipek, An analog preconditioner for solving linear systems, in *2021 IEEE International Symposium on High–Performance Computer Architecture (HPCA)* (IEEE, 2021) pp. 761–774.

57. J. R. Nagel, Numerical solutions to poisson equations using the finite-difference method. *IEEE Antennas Propag. Mag.* **56**, 209–224 (2014).

58. C. Dick, M. Rogowsky, R. Westermann, Solving the fluid pressure poisson equation using multigrid-evaluation and improvements. *IEEE Trans. Vis. Comput. Graph.* **22**, 2480–2492 (2016).

59. Y. Notay, Flexible conjugate gradients. *SIAM J. Sci. Comput.* **22**, 1444–1460 (2000).

60. V. Simoncini, D. B. Szyld, Flexible inner-outer krylov subspace methods. *SIAM J Numer. Anal.* **40**, 2219–2239 (2002).

61. L. Giraud, S. Gratton, X. Pinel, X. Vasseur, Flexible GMRES with deflated restarting. *SIAM J. Sci. Comput.* **32**, 1858–1878 (2010).

62. J. Lee, D. Shin, Y. Kim, H. J. Yoo, A 17.5-fJ/bit energy-efficient analog SRAM for mixed-signal processing. *IEEE Trans. Very Large Scale Integr. (VLSI) Syst.* **25**, 2714–2723 (2017).

63. B. Murmann, ADC performance survey 1997–2021; https://github.com/bmurmann/ADC-survey.

64. Y. Lu, A. Alvarez, C.-H. Kao, J.-S. Bow, S.-Y. Chen, I. W. Chen, An electronic silicon-based memristor with a high switching uniformity. *Nat. Electron.* **2**, 66–74 (2019).

65. A. Haidar, S. Tomov, J. Dongarra, N. J. Higham, Harnessing GPU Tensor Cores for Fast FP16 Arithmetic to Speed up Mixed-Precision Iterative Refinement Solvers in SC18: International conference for high performance computing, networking, storage and analysis. 603–613 (2018).

66. NVIDIA A100 Tensor Core GPU; http://nvidia.com/en–us/data–center/a100/.

67. Y. Lu, W. Liu, in SC23: International conference for high performance computing, networking, storage and analysis. (2023) pp, 1–15.

68. Y. Fang, Z. Yu, Z. Wang, T. Zhang, Y. Yang, Y. Cai, R. Huang, Improvement of HfO_x-based RRAM device variation by inserting ALD TiN buffer layer. *IEEE Electron Device Lett.* **39**, 819–822 (2018).

69. B. Ku, Y. Abbas, A. S. Sokolov, C. Choi, Interface engineering of ALD HfO_2-based RRAM with Ar plasma treatment for reliable and uniform switching behaviors. *J. Alloys Compd.* **735**, 1181–1188 (2018).

70. S. Lee, J. Sohn, Z. Jiang, H.-Y. Chen, H.-S. Philip Wong, Metal oxide-resistive memory using graphene-edge electrodes. *Nat. Commun.* **6**, 8407 (2015).

71. B. Govoreanu, G. S. Kar, Y. Y. Chen, V. Paraschiv, S. Kubicek, A. Fantini, I. P. Radu, L. Goux, S. Clima, R. Degraeve, N. Jossart, O. Richard, T. Vandeweyer, K. Seo, P. Hendrickx, G. Pourtois, H. Bender, L. Altimime, D. J. Wouters, J. A. Kittl, M. Jurczak, 10×10 nm^2 Hf/HfO_x

crossbar resistive RAM with excellent performance, reliability and low-energy operation, in *2011 International Electron Devices Meeting (IEDM)*. (2011) pp. 31.36.31–31.36.34.

72. D. Veksler, G. Bersuker, A. W. Bushmaker, P. R. Shrestha, K. P. Cheung, J. P. Campbell, Switching variability factors in compliance-free metal oxide RRAM. *in 2019 IEEE International Reliability Physics Symposium (IRPS)*. (IEEE, 2019) pp. 1–5.

73. H. Tian, H.-Y. Chen, B. Gao, S. Yu, J. Liang, Y. Yang, D. Xie, J. Kang, T. L. Ren, Y. Zhang, H.-S. Wong, Monitoring oxygen movement by Raman spectroscopy of resistive random access memory with a graphene-inserted electrode. *Nano Lett.* **13**, 651–657 (2013).

74. X.-F. Wang, H. Tian, H.-M. Zhao, T.-Y. Zhang, W.-Q. Mao, Y.-C. Qiao, Y. Pang, Y.-X. Li, Y. Yang, T.-L. Ren, Interface engineering with MoS_2-Pd nanoparticles hybrid structure for a low voltage resistive switching memory. *Small* **14**, 1702525 (2018).

75. H. Y. Lee, Y. S. Chen, P. S. Chen, P. Y. Gu, Y. Y. Hsu, S. M. Wang, W. H. Liu, C. H. Tsai, S. S. Sheu, P. C. Chiang, W. P. Lin, C. H. Lin, W. S. Chen, F. T. Chen, C. H. Lien, M. J. Tsai, Evidence and solution of over-RESET problem for HfO_x based resistive memory with sub-ns switching speed and high endurance, in *2010 International Electron Devices Meeting (IEDM)*. (2010) pp. 19.17.11–19.17.14.

76. H. Y. Lee, P. S. Chen, T. Y. Wu, Y. S. Chen, C. C. Wang, P. J. Tzeng, C. H. Lin, F. Chen, C. H. Lien, M. J. Tsai, Low power and high speed bipolar switching with a thin reactive Ti buffer layer in robust HfO_2 based RRAM, in *2008 IEEE International Electron Devices Meeting (IEDM)*. (IEEE, 2008) pp. 1–4.

77. J. U. Kwon, Y. G. Song, J. E. Kim, S. Y. Chun, G. H. Kim, G. Noh, J. Y. Kwak, S. Hur, C.-Y. Kang, D. S. Jeong, S. J. Oh, J. H. Yoon, Surface-dominated HfO_2 nanorod-based memristor exhibiting highly linear and symmetrical conductance modulation for high-precision neuromorphic computing. *ACS Appl. Mater. Interfaces* **14**, 44550–44560 (2022).

78. J. Wang, L. Li, H. Huyan, X. Pan, S. S. Nonnenmann, Highly uniform resistive switching in HfO_2 films embedded with ordered metal nanoisland Arrays. *Adv. Funct. Mater.* **29**, 1808430 (2019).

79. Q. Xia, J. J. Yang, Memristive crossbar arrays for brain-inspired computing. *Nat. Mater.* **18**, 309–323 (2019).

80. J. Shang, G. Liu, H. Yang, X. Zhu, X. Chen, H. Tan, B. Hu, L. Pan, W. Xue, R.-W. Li, Thermally stable transparent resistive random access memory based on all-oxide heterostructures. *Adv. Funct. Mater.* **24**, 2171–2179 (2014).

81. H. Jiang, L. Han, P. Lin, Z. Wang, M. H. Jang, Q. Wu, M. Barnell, J. J. Yang, H. L. Xin, Q. Xia, Sub-10 nm Ta channel responsible for superior performance of a HfO_2 memristor. *Sci. Rep.* **6**, 28525 (2016).

82. Y.-L. Zhu, K. H. Xue, X. M. Cheng, C. Qiao, J.-H. Yuan, L. Li, X.-S. Miao, Uniform and robust TiN/HfO_2/Pt memristor through interfacial Al-doping engineering. *Appl. Surf. Sci.* **550**, 149274 (2021).

83. Z. Chen, F. Zhang, B. Chen, Y. Zheng, B. Gao, L. Liu, X. Liu, J. Kang, High-performance HfO_x/AlO_y-based resistive switching memory cross-point array fabricated by atomic layer deposition. *Nanoscale Res. Lett.* **10**, 70 (2015).

84. J. Nebhen, M. Ismail, U. Chand, C. Mahata, S. Kim, Demonstration of synaptic and resistive switching characteristics in W/TiO_2/HfO_2/TaN memristor crossbar array for bioinspired neuromorphic computing. *J. Mater. Sci. Technol.* **96**, 94–102 (2022).

85. M. Ismail, C. Mahata, S. Kim, Forming-free Pt/Al_2O_3/HfO_2/$HfAlO_x$/TiN memristor with controllable multilevel resistive switching and neuromorphic characteristics for artificial synapse. *J. Alloys Compd.* **892**, 162141 (2022).

86. Y. Yang, X. Zhu, Z. Ma, H. Hu, T. Chen, W. Li, J. Xu, L. Xu, K. Chen, Artificial HfO_2/TiO_x synapses with controllable memory window and high uniformity for brain-inspired computing. *Nanomaterials* **13**, 605 (2023).

87. Y.-L. Zhu, L.-H. Li, C. Qiao, Y.-T. Zeng, J.-H. Yuan, X.-M. Cheng, X.-S. Miao, Modulation of oxygen transport by incorporating Sb_2Te_3 layer in HfO_2-based memristor. *Appl. Phys. Lett.* **119**, 193503 (2021).

88. Y. Xiao, X. Wu, Y. P. Jin, G. Cao, B. Jiang, S. Ke, C. Ye, Improved artificial synapse performance of Pt/HfO_2/$BiFeO_3$/HfO_2/TiN memristor through N_2 annealing. *Ceram. Int.* **48**, 34584–34589 (2022).

89. C.-H. Huang, W.-C. Chang, J.-S. Huang, S.-M. Lin, Y.-L. Chueh, Resistive switching of Sn-doped In_2O_3/HfO_2 core-shell nanowire: Geometry architecture engineering for nonvolatile memory. *Nanoscale* **9**, 6920–6928 (2017).

90. Y. Zhou, H. Huang, J. Han, K. Chen, C. Ye, Z. Xu, S. Liang, W. Xiong, X. Chen, Z. Song, M. Zhu, Flexible HfO_2/graphene oxide selector with fast switching and high endurance. *IEEE J. Electron Devices Soc.* **7**, 1125–1128 (2019).

91. M. Ismail, C. Mahata, M. Kang, S. Kim, Robust resistive switching constancy and quantum conductance in high-*k* dielectric-based memristor for neuromorphic engineering. *Nanoscale Res. Lett.* **17**, 61 (2022).
